# An adipose lncRAP2-Igf2bp2 complex enhances adipogenesis and energy expenditure by stabilizing target mRNAs

**DOI:** 10.1016/j.isci.2021.103680

**Published:** 2021-12-25

**Authors:** Juan R. Alvarez-Dominguez, Sally Winther, Jacob B. Hansen, Harvey F. Lodish, Marko Knoll

**Affiliations:** 1 Whitehead Institute for Biomedical Research, 455 Main Street, Cambridge, MA, 02142, USA; 2 Department of Biology, University of Copenhagen, Universitetsparken 13, DK2100, Copenhagen, Denmark; 3 Departments of Biology and Biological Engineering, Massachusetts Institute of Technology, 21Ames Street, Cambridge, MA02142, USA; 4 Department of Cell and Developmental Biology, University of Pennsylvania Perelman School of Medicine, Philadelphia, PA19104, USA; 5 Institute for Diabetes, Obesity and Metabolism, University of Pennsylvania Perelman School of Medicine, Philadelphia, PA19104, USA; 6 Institute for Diabetes Research, Helmholtz Zentrum München, Heidemannstrasse 1, 80939München, Germany

**Keywords:** Biological sciences, Molecular physiology, Molecular biology, Omics

## Abstract

lncRAP2 is a conserved cytoplasmic lncRNA enriched in adipose tissue and required for adipogenesis. Using purification and *in vivo* interactome analyses, we show that lncRAP2 forms complexes with proteins that stabilize mRNAs and modulate translation, among them Igf2bp2. Surveying transcriptome-wide Igf2bp2 client mRNAs in white adipocytes reveals selective binding to mRNAs encoding adipogenic regulators and energy expenditure effectors, including adiponectin. These same target proteins are downregulated when either Igf2bp2 or lncRAP2 is downregulated, hindering adipocyte lipolysis. Proteomics and ribosome profiling show this occurs predominantly through mRNA accumulation, as lncRAP2-Igf2bp2 complex binding does not impact translation efficiency. Phenome-wide association studies reveal specific associations of genetic variants within both lncRAP2 and Igf2bp2 with body mass and type 2 diabetes, and both lncRAP2 and Igf2bp2 are suppressed in adipose depots of obese and diabetic individuals. Thus, the lncRAP2-Igf2bp2 complex potentiates adipose development and energy expenditure and is associated with susceptibility to obesity-linked diabetes.

## Highlights

• lncRAP2 is a cytosolic lncRNA conserved in mouse and human needed for adipogenesis
• lncRAP2 complexes with mRNA stability and translation regulators, including Igf2bp2
• lncRAP2-Igf2bp2 stabilizes lipid metabolism mRNAs to potentiate energy expenditure
• lncRAP2-Igf2bp2 genetic and expression variation is linked to BMI, type 2 diabetes

## Graphical abstract

**Figure:**
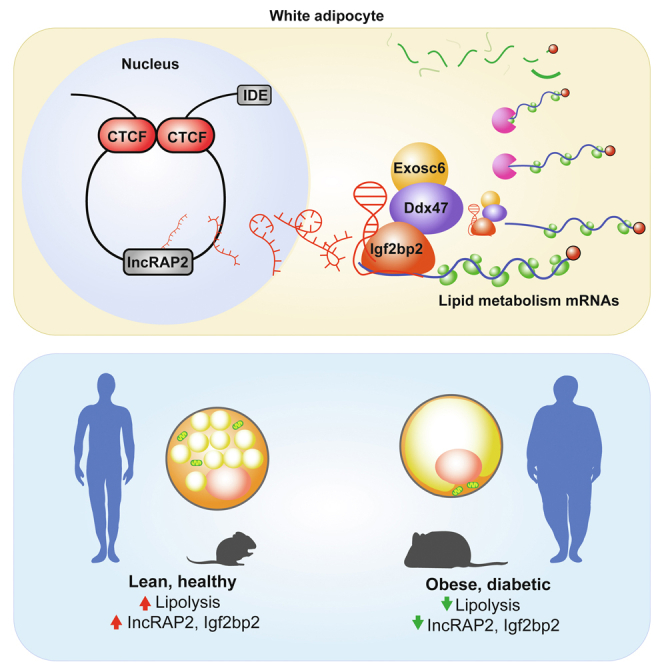

## Introduction

Pervasive transcription of the human and mouse genomes generates thousands of long noncoding RNAs (lncRNAs), but only a small minority have been linked to specific biochemical functions. Previous studies revealed that many lncRNAs are specifically enriched in white and/or brown adipocytes and play vital roles in adipocyte biology (Knoll et al., 2015; Sun and Lin, 2019), including in adipogenesis, thermogenesis, and insulin sensitivity. As example, we and others showed that the brown fat-specific lncRNAs Blnc1 (Zhao et al., 2014) and lncBATE1 (Alvarez-Dominguez et al., 2015) interact with the nuclear matrix factor hnRNPU to mediate *trans*-activation of genes mediating brown and/or beige thermogenic programs.

While lncRNAs can potentially bind DNA, RNA, or protein targets, much work suggests that lncRNA mechanisms predominantly involve binding to proteins, either as scaffolds for ribonucleoprotein complexes or as decoys that prevent their assembly (Alvarez-Dominguez and Lodish, 2017; Wang and Chang, 2011). To better understand how lncRNAs function, several technologies have been recently developed for unbiased determination of lncRNA localization, protein targets, and functional domains (Goff and Rinn, 2015; McDonel and Guttman, 2019). Single-molecule fluorescence *in situ* hybridization (smFISH) visually determines RNA abundance and location, revealing whether a lncRNA diffuses to *trans* sites beyond its chromosomal locus or remains tethered *in cis* in the nucleus (Cabili et al., 2015; Raj et al., 2008). Cross-linking intact cells followed by hybridization-based RNA purification captures the specific DNA, RNA, or protein targets to which a lncRNA binds *in vivo* (rather than in solution after cell lysis) (Chu et al., 2011, 2015; Engreitz et al., 2013; Simon et al., 2013). And footprint profiling can map the RNA sequence sites where proteins bind lncRNAs, revealing their functional domains (Darnell, 2010; Ingolia et al., 2009; Silverman et al., 2014).

Here, we use these and other tools to interrogate the mode of action of lncRAP2, a white adipocyte-selective RNA that is essential for adipogenesis (Sun et al., 2013). We show that lncRAP2 predominantly resides in the cytoplasm, yet it does not directly associate with ribosomes or other RNAs. Instead, lncRAP2 forms a complex with several RNA-binding proteins that affect mRNA stability and translation. Among these is Igf2bp2, which has been implicated in posttranscriptional control of metabolically important proteins (Dai et al., 2015; Laggai et al., 2014; Zhang et al., 2018). We identify the transcriptome-wide Igf2bp2 mRNA clients in white adipocytes, which include key adipogenic effectors and mediators of energy metabolism. Indeed, we show that depleting either lncRAP2 or Igf2bp similarly downregulates the proteins encoded by these targets, including adiponectin, and that this occurs primarily through mRNA destabilization. Accordingly, adipocytes in which either lncRAP2 or Igf2bp is depleted show compromised energy expenditure. We further find that the levels of lncRAP2 and Igf2bp2 in adipocytes are reduced during the development of obesity and diabetes. Analysis of genome-wide association studies reveals a specific association of lncRAP2 and Igf2bp2 polymorphic alleles with increased body fat and greater risk of type 2 diabetes. Thus, a previously uncharacterized lncRAP2-Igf2bp2 complex regulates adipose energy expenditure, with implications for the susceptibility to and pathogenesis of obesity-linked diabetes in humans.

## Results

### lncRAP2 is a conserved, adipose-enriched cytoplasmic lncRNA required for adipogenesis

**Figure 1:**
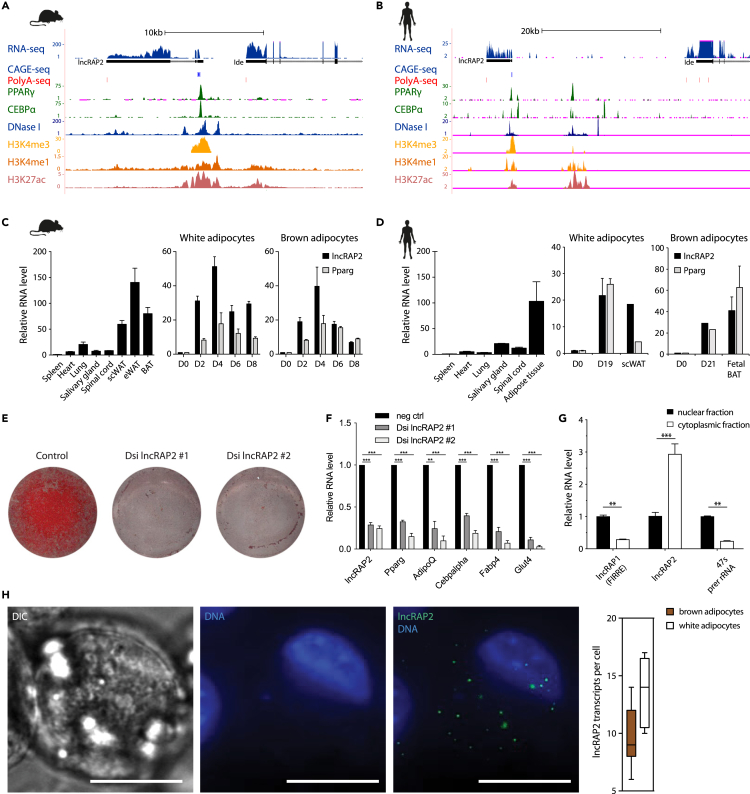
lncRAP2 is a conserved cytoplasmic RNA required for adipogenesis (A and B) lncRAP2 is a capped, polyadenylated, and spliced RNA transcribed from a promoter bound by PPARγ and C/EBPα. Tracks show signal from sequencing studies of white adipocytes from mouse (A) and human (B) (Data S4). (C and D) lncRAP2 is adipocyte-specific and strongly induced during adipogenesis. Shown are relative tissue expression (normalized to spleen, left) and relative induction during *in vitro* differentiation of white (center) or brown (right) preadipocytes from mouse (C) and human (Ding et al., 2018) (D). scWAT, subcutaneous adipose tissue; eWAT, epididymal white adipose tissue; BAT, brown adipose tissue. (E and F) lncRAP2 depletion blocks adipogenesis. Shown are lipid accumulation by Oil-red O staining (E) and relative expression of key adipocyte genes (F) in day 6 differentiated white adipocytes pretreated with control or two different lncRAP2-targeting DsiRNAs. (G and H) lncRAP2 localizes to the cytoplasm. Relative expression in fractionated nuclear and cytoplasmic compartments (G) and single-molecule FISH detection (H) in day 6 differentiated white adipocytes. lncRAP2 molecules above nucleus overlay DAPI staining in maximum z stack projections of FISH images, quantified to the right (n = 9 cells with ≥1 transcripts). ∗∗p <0.01, ∗∗∗p <0.001 (ttest).

We previously identified several lncRNAs common to white and brown adipose that, based on effects of depleting them in mouse preadipocytes, are essential for adipocyte development and function (Sun et al., 2013). One of them, lncRAP2 (GenBank: NR_040299.1), is transcribed from an intergenic promoter bound by PPARγ, C/EBPα, and C/EBPβ (Figures 1A and S1A). 5’/3′ RACE verifies a capped and polyadenylated, 6.8kb spliced RNA (Figure S1B). lncRAP2 is highly adipose tissue-specific and strongly induced during early white and brown adipogenesis (Figure 1C). Notably, lncRAP2's structure, regulation, and expression traits are conserved in humans (Figures 1B and 1D). Depleting lncRAP2 by ∼70%–75% by transducing Dicer-substrate siRNAs (DsiRNAs) into primary white preadipocytes (Isidor et al., 2016) dramatically blocks their subsequent differentiation in culture, as evidenced by impaired lipid accumulation and blunted induction of key adipocyte genes (PPARγ, C/EBPα, Adiponectin, Fabp4, and Glut4) (Figures 1E and 1F). Single-molecule fluorescence *in situ* hybridization (smFISH) reveals that lncRAP2 diffuses from the nucleus, spreading throughout the cytoplasm at 14 ± 3 transcripts per white and 9 ± 3 transcripts per brown adipocyte (Figure 1H). Cell fractionation verifies its cytoplasmic localization, unlike lncRAP1 (also called FIRRE) (Figure 1G), which mediates nuclear *trans*-chromosomal interactions among adipogenic genes (Hacisuleyman et al., 2014). These results show that lncRAP2 is a conserved cytoplasmic RNA essential for adipogenesis.

### lncRAP2 forms complexes with proteins that regulate mRNA stability and translation, including Igf2bp2

**Figure 2:**
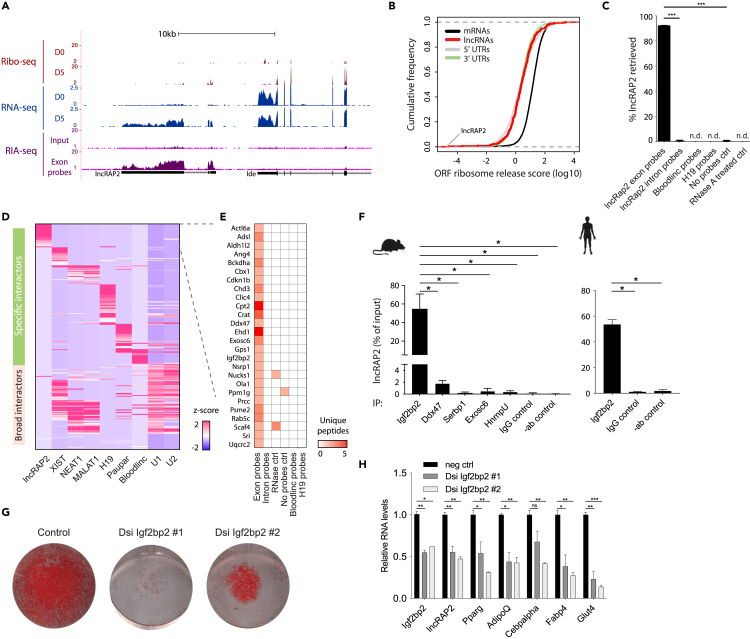
lncRAP2 forms a complex with mRNA stability and translation regulators (A and B) lncRAP2 does not engage translating ribosomes. Tracks show signal from ribosome profiling, RNA, and RNA interactome sequencing studies of differentiated white adipocytes (A). Data are pooled from n = 2–3 replicates. Translatability, measured by ribosome release from open reading frames after encountering a stop codon, is quantified in (B). (C) Efficient and specific enrichment of mature lncRAP2 by hybridization-based purification. Exon-targeting probes effectively retrieve ∼90% of cellular lncRAP2 RNA, quantified by qPCR, from differentiated 3T3-L1 adipocytes, whereas <1% was retrieved by intron-targeting probes, no probes, or probes targeting unrelated RNAs. RNase treatment eliminates lncRAP2 transcripts prior to purification. ∗∗∗p <0.001 (t test). (D and E) ChIRP-MS identifies specific lncRAP2-binding proteins. The relative enrichment of high-confidence interactors captured by antisense purification of lncRAP2 in white adipocytes or other lncRNAs in other formaldehyde cross-linked cells is shown in (D). Unique peptide counts for specific lncRAP2 interactors are shown in (E). (F) Validation of lncRAP2 and Igf2bp2 direct interaction in mature white adipocytes from mouse (left) and human (right). Native immunoprecipitation of Igf2bp2 specifically captures >50% of lncRAP2, compared to <2% for Ddx47 and Exosc6 (specific interactors) or Serbp1 and HnrpU (broad interactors). ∗p <0.05 (t test). (G and H) Igf2bp2 depletion blocks adipogenesis. Shown are lipid accumulation (G) and relative expression of key adipocyte genes (H) in day 6 differentiated white adipocytes pretreated with control or Igf2bp2-targeting DsiRNAs.

To investigate how lncRAP2 functions, we sought to identify its binding partners and targets. lncRAP2's mRNA-like features suggest that it could bind ribosomes, which we tested by examining parallel RNA and ribosome footprint profiling during mouse adipogenesis (Reid et al., 2017). Despite strong induction during adipogenesis, lncRAP2 is largely devoid of ribosome-protected RNA fragments, and exhibits poor translatability (Figures 2A and 2B). Supporting the notion that lncRAP2 is not translated, no peptides could be found in proteome surveys of brown or white adipocytes from either mouse or human (Alvarez-Dominguez et al., 2015; Desiere et al., 2006). We conclude that lncRAP2 is unlikely to engage translating ribosomes.

To identify lncRAP2's binding targets, we used biotin-labeled smFISH probes to purify lncRAP2 from cross-linked intact mouse 3T3-L1 adipocytes, which express lncRAP2 at levels comparable to those of primary adipocytes (Figure S2A). Using exon-targeting probes, we retrieved ∼90% of cellular lncRAP2, whereas less than 1% was retrieved following RNase A treatment, without targeting probes, or with probes targeting introns or unrelated lncRNAs like Bloodlinc (an erythrocyte-specific lncRNA) (Alvarez-Dominguez et al., 2014) or H19 (a broadly expressed lncRNA) (Pachnis et al., 1984) (Figure 2C). These controls attest to the efficiency and specificity of our RNA antisense purification protocol. We then used RNA interactome analysis by sequencing (RIA-seq) (Kretz et al., 2013) to probe interactions of lncRAP2 with other RNAs *via* glutaraldehyde cross-linking. lncRAP2 was robustly retrieved by distinct pools of exon-targeting probes, but no other RNAs were cross-linked with significant enrichment or with concordance between probe pools (Figures S2B and S2C), indicating that lncRAP2 does not directly bind other RNAs.

We then conducted a comprehensive identification of RNA-binding proteins by mass spectrometry (ChIRP-MS) (Chu et al., 2015) in 3T3-L1 adipocytes using formaldehyde cross-linking, which preserves both direct and indirect RNA-protein interactions. As controls, we compared cross-linked proteins to those captured using RNase A treatment, intron-targeting probes, non-targeting probes, or no probes. lncRAP2 purifications retrieved rich protein analytes compared to these controls (Figure S2D), capturing 621 proteins detected by at least 2 unique peptides. We found 29 of these to be high-confidence (>2-fold enriched) lncRAP2-binding proteins (Data S1). To identify specific lncRAP2 interactors, we compared these proteins to those captured by the hybridization-based purification of other lncRNAs: Xist, Neat1, Malat1, H19, Paupar, and Bloodlinc (Alvarez-Dominguez et al., 2017a; Chu et al., 2015; Schmidt et al., 2018; Singer et al., 2019; West et al., 2014) in formaldehyde cross-linked cells. Notably, 26 out of the 29 interactors (90%) are lncRAP2-specific (Figures 2D and 2E), and include regulators of fatty acid and keto acid metabolism (Crat, Cpt2, and Bckdha) as well as regulators of mRNA translation and decay (e.g. Igf2bp2, Exosc6, and Ddx47).

Among lncRAP2 interactors, Igf2bp2 regulates adipocyte function *via* posttranscriptional control of metabolically important proteins, impacting sensitivity to diet-induced obesity and type 2 diabetes risk (Dai et al., 2015; Laggai et al., 2014; Zhang et al., 2018). Native immunoprecipitation of endogenous Igf2bp2 in mouse and human white adipocytes specifically captures over 50% of lncRAP2 (Figures 2F and S2E), which verifies direct lncRAP2-Igf2bp2 interactions. Igf2bp2 forms ribonucleoprotein complexes with client mRNAs to modulate their stability and translation (Dai et al., 2011; Hafner et al., 2010; Nielsen et al., 1999). Our data indicate that such complexes include Ddx47, which is thought to perform RNA unwinding during mRNA translation and decay (Hock et al., 2007), and Exosc6/Mtr3, which binds to and presents mRNAs for degradation by the exosome (Chen et al., 2001). Native immunoprecipitation of Ddx47 or Exosc6, however, captures less than 2% of cellular lncRAP2 (Figure 2F), suggesting an indirect association to lncRAP2 *via* protein intermediates. We thus focused on the lncRAP2-Igfb2p connection.

### lncRAP2 and Igf2bp2 stabilize target mRNAs encoding metabolic effectors to potentiate adipocyte energy expenditure

Igf2bp2, like lncRAP2, is highly expressed in white adipocytes (Figure S2F). We find little Igf2bp2 induction with adipogenesis, however, as it is already present in pre-adipocytes (Figures S2G and S2H). Depleting *Igf2bp2* by ∼50% with DsiRNAs in white preadipocytes blocks their subsequent differentiation, analogous to lncRAP2 inhibition, blunting induction of adipogenic markers, and lipogenesis (Figures 2G and 2H). The induction of lncRAP2 is also disrupted, consistent with a block in differentiation.

**Figure 3:**
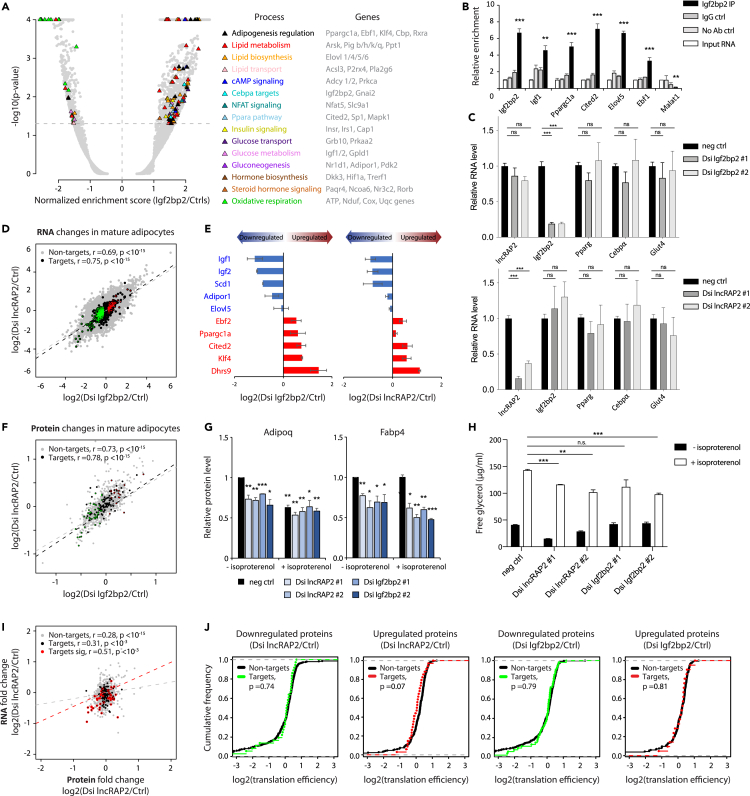
lncRAP2-Igf2bp2 complex regulates expression of target mRNAs encoding metabolically important proteins to potentiate adipocyte energy expenditure (A and B) Igf2bp2 selectively binds mRNAs encoding adipogenic regulator and effector proteins in mouse white adipocytes. Shown is a gene set enrichment analysis highlighting significantly enriched (p <0.05) gene sets and their associated biological processes (A), with member genes shown to the right, and specific enrichment of mRNAs encoding key energy metabolism controllers (B), from native Igf2bp2 immunoprecipitations. ∗∗p <0.01, ∗∗∗p <0.001 (ttest). (C) Depleting lncRAP2 or *Igf2bp2* in mature adipocytes does not alter expression of adipose marker genes or each other's RNA levels. Relative expression of key adipocyte genes in day 6 differentiated white adipocytes pretreated at differentiation day 4 with control, Igf2bp2-targeting (top), or lncRAP2-targeting (bottom) DsiRNAs. (D and E) RNA changes after *Igf2bp2* depletion in mature adipocytes correspond to those after lncRAP2 depletion. RNA changes for Igf2bp2 targets (black circles) and non-targets (gray circles) are shown in (D). Targets that are upregulated (red) or downregulated (green) after *Igf2bp2* depletion (p <0.05, ttest) are highlighted. Changes for select targets are shown in (E). (F and G) Protein changes after *Igf2bp2* depletion in mature adipocytes correspond to those after lncRAP2 depletion. Protein changes for Igf2bp2 targets (black circles) and non-targets (gray circles) are shown in (F). Targets that are upregulated (red) or downregulated (green) after *Igf2bp2* depletion (p <0.05, ttest) are highlighted. Changes for select targets in isoproterenol-stimulated or non-stimulated mature adipocytes are shown in (G). ∗p <0.05, ∗∗p <0.01, ∗∗∗p <0.001 relative to negative control (t test). (H) lncRAP2-Igf2bp2 potentiates energy expenditure. Lipolysis assays measuring glycerol release in isoproterenol-stimulated or non-stimulated lncRAP2 or *Igf2bp2* -depleted white adipocytes are shown. ∗p <0.05, ∗∗p <0.01, ∗∗∗p <0.001 relative to negative control (ttest). (I) Correspondence between RNA and protein changes upon lncRAP2 depletion in mature adipocytes, for 1,205 genes quantified in both transcriptomic and proteomic datasets. Igf2bp2 targets (black circles) and non-targets (gray circles) are shown. Targets with differentially regulated protein levels (p <0.05, ttest) are highlighted in red. (J) The translation efficiency of Igf2bp2 target mRNAs is indistinct from that of non-targets. Translation efficiency, measured by the enrichment of ribosome footprint profiling over RNA-seq reads in visceral adipose tissue (Reid et al., 2017), is shown for Igf2bp2 targets and non-targets upregulated (red) or downregulated (green) at the protein level upon lncRAP2 (left panels) or *Igf2bp2* (right panels) depletion in mature white adipocytes.

To investigate how Igf2bp2 regulates adipogenesis, we sought to identify its client RNAs. Unbiased sequencing of RNAs captured by native Igf2bp2-specific immunoprecipitates reveals 1,657 substrates enriched over input RNA or control purifications with IgG or with no antibody (Figure S3A; Data S2). These comprise ncRNAs and mRNAs linked to various functions, with selective enrichment for adipogenic effectors (e.g. Nfat and Elovl proteins) and regulators (e.g. Cbp, Cited2, Ebf, and Klf factors) (Figure 3A). In line with known Igf2bp2 binding bias (Conway et al., 2016; Hafner et al., 2010; Ray et al., 2013), >60% of Igf2bp2 targets in adipocytes harbor CA-rich binding motifs, mainly enriched in 3′UTRs (Figure S3B), including several motif instances within lncRAP2's terminal exon (Figure S3C). Of 1,477 Igf2bp2 substrates with human homologs, 1,095 (∼74%) also copurify with Igf2bp2 in human cell types cross-linked with UV (Van Nostrand et al., 2016) or 4-thiorudine (Hafner et al., 2010) (Figure S3D). Notably, conserved substrates include mRNAs encoding key controllers of energy metabolism, such as the namesake Igf1/2 targets and PPARγ coactivator 1α (PGC1α) (Figure 3B).

Igf2bp2 client RNAs that are induced or suppressed with adipogenesis are reciprocally regulated if lncRAP2 is depleted before differentiation (Sun et al., 2013) (Figure S3E). This could reflect Igf2bp2 and lncRAP2 sharing a common set of RNA targets, or merely the fact that both affect adipogenesis. To distinguish between these possibilities, we depleted lncRAP2 and Igf2bp2 in mature adipocytes, which allow studying the lncRAP2-Igfb2p connection independent of effects on adipogenesis. Transducing DsiRNAs against lncRAP2 or *Igf2bp2* into differentiated white adipocytes deplete their target by 70%–80% without impactingeach other's levels (Figure 3C). Remarkably, unbiased RNA sequencing reveals that the RNA changes occurring after lncRAP2 depletion are tightly correlated with those occurring after Igf2bp2 depletion (Pearson's r = 0.75, p <10^−15^, t test) (Figures 3D and S3F). By contrast, we find no correlation (Pearson's r ∼0) between the global RNA effects of depleting lncRAP2 or *Igf2bp2* with those of depleting *Pparγ* or *linc-ADAL* (lincRNA for adipogenesis and lipogenesis) in mature white adipocytes (Schupp et al., 2009; Zhang et al., 2018) (Figure S3H). Inhibiting either *Igf2bp2* or lncRAP2 results in destabilization of the namesake Igf1/2 mRNA targets of Igf2bp2, as well as derepression of target mRNAs encoding thermogenic factors Ebf2 and PGC1α (Figure 3E). By contrast, non-target mRNAs encoding key adipogenic regulators (PPARγ, C/EBPα) remain unaffected (Figure 3C).

Igf2bp2 client mRNAs encoding effectors of lipid/glucose synthesis and metabolism are selectively destabilized when either lncRAP2 or *Igf2bp2* is inhibited (Figure S3G), suggesting that the lncRAP2-Igf2bp2 complex normally potentiates energy expenditure. To test this, we measured lipolysis in differentiated lncRAP2- and *Igf2bp2*-depleted adipocytes. In both cases, isoproterenol-induced lipolytic responses were reduced, and lncRAP2 depletion also lowered basal lipolysis (Figure 3H). Further supporting key roles in lipid metabolism, the gene signature of lncRAP2/*Igf2bp2* depletion is most significantly associated with that of lysosomal acid lipase deficiency in lysosomal acid lipase gene knockout mice (Lian et al., 2005) (Figure S3I). These findings indicate that lncRAP2-Igf2bp2 complexes support energy expenditure in mature adipocytes by binding to and stabilizing many mRNAs encoding metabolic effectors.

### lncRAP2 and Igf2bp2 predominantly modulate target mRNA levels, not their translation

To investigate how lncRAP2 and Igf2bp2 regulate their mRNA targets, we studied their protein levels and mRNA translation efficiency. Global protein levels were examined by quantitative mass spectrometry using tandem mass tag labeling in mature adipocytes. In total, proteins for 1,273 genes were quantifiable (detected by at least 2 unique peptides), including 138 whose mRNAs were found to be direct Igf2bp2 targets by RIP-seq. For both direct Igf2bp2 targets and non-targets, the changes in protein levels occurring upon lncRAP2 depletion closely correspond to those occurring upon *Igf2bp2* depletion (Pearson's r = 0.78 and r = 0.73, p <10^−15^, t test) (Figure 3F). These results support the notion that an lncRAP2-Igf2bp2 complex regulates a common set of mRNA targets. The targets include proteins with roles in lipid/glucose metabolism and oxidation that are selectively destabilized when either lncRAP2 or *Igf2bp2* is depleted (Figure S4A), such as adiponectin and Fabp4, which further decrease under isoproterenol-induced stimulation of lipolysis (Figure 3G). By contrast, Igf2bp2 protein abundance was unaltered by lncRAP2 depletion, consistent with RNA-level results indicating that lncRAP2 and Igf2bp2 do not act to regulate each other's levels.

The closely corresponding RNA and protein changes after either lncRAP2 or Igf2bp2 are inhibited suggests that the lncRAP2-Igf2bp2 complex regulates its targets by tuning their mRNA and thereby protein level. To test this, we compared RNA and protein responses to lncRAP2 or *Igf2bp2* depletion in mature adipocytes for 1,205 genes quantifiable by both RNA-seq and mass spectrometry. The correlation between the two types of responses was strong for direct Igf2bp2 targets with differentially regulated protein vs. non-targets, both after lncRAP2 depletion (Pearson's r = 0.51 vs. r = 0.28, p <10^−3^ vs. p <10^−15^, t test) and after Igf2bp2 depletion (Pearson's r = 0.32 vs. r = 0.12, p <10^−1.3^ vs. p <10^−3^, t test) (Figures 3I and S4B). Any scatter that might have indicated that a few targets are translationally regulated (changing in protein but not RNA level) closely resembled the scatter observed in parallel for non-targets. We also calculated mRNA translation efficiencies, by dividing the level of steady-state ribosome-protected RNA fragments by that of total RNA fragments measured in parallel in mature adipocytes (Reid et al., 2017). We found no significant difference in translation efficiency between direct Igf2bp2 targets and non-targets, whether down/up regulated at the protein level after lncRAP2/*Igf2bp2* depletion (Figure 3J). In summary, we found no evidence countering the conclusion that lncRAP2-Igf2bp2 complexes mainly act to modulate mRNA levels for most targets, without directly affecting their translation.

### lncRAP2 and Igf2bp2 are associated with obesity-linked diabetes risk

**Figure 4:**
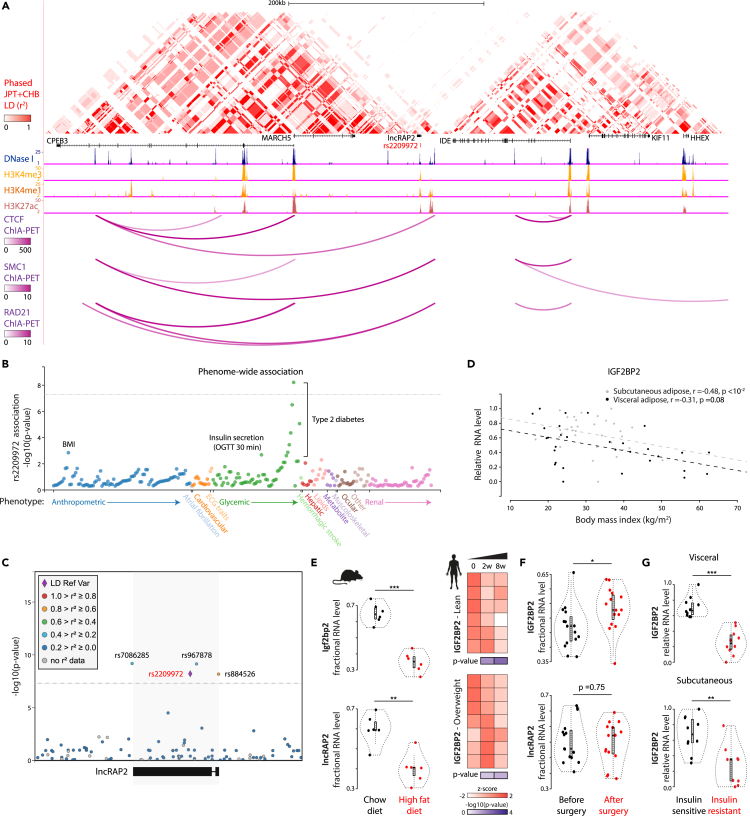
lncRAP2-Igf2bp2 genetic and expression variability are associated with obesity-linked diabetes risk (A) A genetic variant linked to higher body mass, fasting insulin, and insulin resistance in Chinese females (Wang et al., 2008) (rs2209972) maps to a lncRAP2 linkage disequilibrium and structural domain. Heatmap displays linkage disequilibrium (LD) for a population of Han Chinese in Beijing and Japanese in Tokyo (JPT + CHB) (International HapMap, 2003) from phased genotypes of 90 unrelated individuals (Barrett et al., 2005). Tracks below display human white adipocyte open chromatin and histone mark sequencing studies, and chromatin interactions involving CTCF and cohesin subunits (SMC1, RAD21) (Data S4). (B) lncRAP2rs2209972 is specifically associated with body mass, insulin secretion, and type 2 diabetes. Phenome-wide association results between rs2209972 and 317 phenotypes across 16,278,030 individuals of various ancestries from the Type 2 Diabetes Knowledge Portal (http://www.type2diabetesgenetics.org/variantInfo/variantInfo/rs2209972). Only significant (p <0.05) associations are shown. (C) lncRAP2 locus-wide association with type 2 diabetes. Significance of association between genetic variants within and around the lncRAP2 locus (highlighted) and type 2 diabetes in 898,130 European-descent individuals (Mahajan et al., 2018). Variants are colored based on 1000 Genomes All samples (Genomes Project et al., 2015) LD with rs2209972 (diamond). (D and E) Adipose lncRAP2 and *Igf2bp2* are progressively suppressed with obesity. Body mass index vs. *Igf2bp2* levels in visceral (Barberio et al., 2019) (black) or subcutaneous (Keller et al., 2011) (gray) adipose tissue from 63 individuals are shown in (D). Relative lncRAP2 and *Igf2bp2* expression in mice (Jones et al., 2020) (visceral fat, left) or lean/overweight humans (Alligier et al., 2012) (subcutaneous fat, right) fed a high-fat diet are shown in (E). (F) Adipose lncRAP2 and *Igf2bp2* are restored with weight loss after bariatric surgery. Relative lncRAP2 and *Igf2bp2* expression in subcutaneous adipose tissue samples from 15 obese women before and 3 months after Roux-en-Y gastric bypass surgery (Poitou et al., 2015). (G) Adipose *Igf2bp2* is suppressed with insulin resistance. Relative *Igf2bp2* expression in both visceral (top) and subcutaneous (bottom) adipose tissue samples from 10 insulin-sensitive or 10 insulin-resistant body mass index-matched obese patients undergoing gastric bypass surgery (Hardy et al., 2011). ∗p <0.05, ∗∗p <0.01, ∗∗∗p <0.001 (t test).

Given lncRAP2's role in regulating adipocyte development and function, we sought validation of its importance from human genetics. A genetic variant (rs2209972:C) associated with increased body mass index, higher fasting insulin levels, and insulin resistance in women with polycystic ovary syndrome in a Chinese population (Wang et al., 2008) maps to the lncRAP2 gene body (Figure 4A). While this variant has been associated with the nearby gene encoding insulin-degrading enzyme (IDE) (Wang et al., 2008), three lines of evidence confine its genetic association to the lncRAP2 locus. First, in the Chinese and in all other populations surveyed, rs2209972 is in strong linkage disequilibrium (r^2^ ≥ 0.8) with variants within lncRAP2, but not so with any other locus within 500kb, including the IDE gene (Figures 4A and S5A). Second, lncRAP2 is confined to a closed chromatin loop formed by interacting sites of the CTCF insulator that are co-bound by cohesin (Figure 4A), insulating the lncRAP2 and IDE genes from each other. Third, rs2209972 and linked variants physically contact lncRAP2 but no loci outside its insulated structural domain, as evidenced by chromatin conformation analysis (Figure S5B).

To probe rs2209972 phenotype associations in an unbiased manner, we examined 99 genome-wide association studies of 234 human traits. These included traits relevant to metabolic, cardiovascular, hepatic, neural, renal, and musculoskeletal disorders, as well as anthropometric traits. Our analysis revealed specific associations with body mass index, insulin secretion, and type 2 diabetes (Figure 4B; Table 1). Of these associations, increased diabetes risk in individuals of European ancestry was the strongest (odds ratio = 1.04 per rs2209972:C allele, p <10^−8^). Additional genetic variants mapping to the lncRAP2 transcription start and end sites (rs884526 and rs7086285, respectively) and gene body (rs967878) also represent risk alleles for type 2 diabetes (p < 10^9^ to 10^−8^), thus evidencing association of multiple lncRAP2 alleles with diabetes (Figure 4C; Table 1). Notably, the Igf2bp2 gene is also specifically associated with the same metabolic traits (Figures S5C and S5D), in line with lncRAP2-Igf2bp2 complexes modulating common pathways.

**Table 1:** Reported variants and their associated traits

Variant	Trait	p value	Effect direction	Odds ratio	Effect size	Sample size
rs2209972(C)	Type 2 diabetes	6.1E-09	Increased	1.039		898,130
	BMI	0.00142	Increased		0.0132	191,764
	Incremental insulin at 30 min OGTT	0.00207	Decreased		−0.0800	5,318
rs967878(G)	Type 2 diabetes	7.10E-10	Increased	1.039		898,130
rs7086285(T)	Type 2 diabetes	6.50E-10	Increased	1.039		898,130
rs884526(G)	Type 2 diabetes	6.60E-09	Increased	1.039		898,130
	BMI	0.00136	Increased		0.0133	191,764

The pathogenesis of type 2 diabetes can involve obesity-linked adipose dysfunction (Ashcroft and Rorsman, 2012; Rosen and Spiegelman, 2006). To explore lncRAP2-Igf2bp2's contribution, we studied their regulation in obesity and diabetes progression. Adipose tissue lncRAP2/*Igf2bp2* levels are progressively suppressed in mice and in lean/overweight humans fed a high-fat diet (p <10^−3^ to 10^−1.3^, t test) (Figure 4E). Human adipose lncRAP2/*Igf2bp2* levels decrease as body mass index increases (Figures 4D and S5E), and tend to be restored upon weight loss after bariatric surgery (Figure 4F). We also found that *Igf2bp2* trends lower in adipocytes of leptin-deficient (ob/ob) and leptin receptor-deficient (db/db) obese mice (Figure S5F), which model diabetes onset and progression (Kleinert et al., 2018). We specifically studied *Igf2bp2* in advanced human diabetics, matched for body mass index, and found strong downregulation in both visceral and subcutaneous adipose tissue of insulin-resistant compared with insulin-sensitive patients (p <10^−3^ to 10^−2^, ttest) (Figure 4G). Thus, both genetic and expression variation of lncRAP2-Igf2bp2 are associated with obesity-linked diabetes outcomes. We propose that this results from reduced levels of lncRAP2-Igf2bp2 complexes, which limits their ability to program adipocyte metabolism by stabilizing mRNAs that encode key energy expenditure proteins.

## Discussion

Obesity has become pandemic (Hales et al., 2018; Vardell, 2020), increasing the worldwide prevalence of type 2 diabetes (Organization, 2016). Anti-diabetic drugs targeting white fat have not been successful (Kusminski et al., 2016; Soccio et al., 2014), highlighting limited insight into how adipocytes develop and function. We and others have shown that lncRNAs contribute to adipocyte lineage specification and specialization (Knoll et al., 2015; Sun and Lin, 2019). Elucidating specific mechanisms has been elusive, however, due to the poor conservation of lncRNA genes and to the scarcity of lncRNA-centric tools. In this study, we made three important contributions to understanding how lncRAP2, conserved between mice and humans, regulates adipocyte function. First, multiple lncRAP2 alleles are specifically associated with diabetes, obesity, and metabolic traits. Second, lncRAP2 forms complexes with the RNA-binding protein Igf2bp2. And third, lncRAP2-Igf2bp2 complexes regulate metabolism in mature adipocytes by stabilizing client mRNAs encoding effectors of energy metabolism.

Our data show that multiple genetic variants associated with propensity to type 2 diabetes are confined to an lncRAP2 linkage disequilibrium and chromatin interaction domain. Analyzing hundreds of phenotypes interrogated by genome-wide association studies reveals that these variants, mapping to the lncRAP2 gene body and transcription start and end sites, are specifically associated with body fat mass, insulin secretion, and type 2 diabetes, implicating lncRAP2 alleles in the development of obesity and diabetes. Supporting this notion, lncRAP2 is suppressed in the white fat of mice and humans consuming a high-fat diet, and in humans lncRAP2 levels decrease progressively with increasing obesity, whereas lncRAP2 levels are restored upon weight loss.

lncRAP2 is enriched in white relative to brown adipocytes and is critical for adipogenesis (Sun et al., 2013). Although many lncRNAs appear to be nuclear-enriched (Derrien et al., 2012), lncRAP2 is mainly cytoplasmic, yet by ribosome footprint profiling we find no evidence of productive lncRAP2 translation. Using RNA interactome analyses in intact cross-linked cells, we found that lncRAP2 does not directly bind other RNAs, but specifically interacts with metabolic enzymes and mRNA decay/translation modulators. Some of these appear to be transient or indirect interactions, which may be of importance in other tissues where lncRAP2 is expressed. Importantly, in both mouse and human adipocytes, we verified a strong, direct binding of lncRAP2 to Igf2bp2.

Igf2bp2 is of prime interest, as it harbors one of the first genetic variants associated by genome-wide association studies with type 2 diabetes (Diabetes Genetics Initiative of Broad Institute of et al., 2007;Scott et al., 2007; Zeggini et al., 2007). This variant, and others in strong linkage disequilibrium, are associated with lower *Igf2bp2* levels in pancreatic islets (Greenwald et al., 2019), and with impaired insulin responses (Wood et al., 2017). Yet the variants are also associated with increased body fat (Pulit et al., 2019), with a stronger effect in type 2 diabetes (Akiyama et al., 2017). Intriguingly, Igf2bp2-null mice are resistant to diet-induced obesity and diabetes, though this is likely due to underdeveloped white and overactive brown fat (Dai et al., 2015)—a tissue that is much less prevalent in adult humans (Rosen and Spiegelman, 2014). How Igf2bp2 impacts diet-induced obesity and diabetes risk, and its roles in mature white fat, have thus remained unclear.

We find that, in mature white adipocytes, Igf2bp2 binds to mRNAs encoding key adipogenic regulators and regulates their stability, explaining why Igf2bp2 is required for adipogenesis *in vitro* and *in vivo* (Dai et al., 2015; Zhang et al., 2018). Igf2bp2 also binds to mRNAs encoding effectors of energy metabolism, including Elovl factors, Fabp4, and adiponectin, thereby linking Igf2bp2 to lipid synthesis, transport, and metabolism. Indeed, we find hindered lipolytic responses upon *Igf2bp2* inhibition, suggesting reduced energy expenditure capacity. Notably, Igf2bp2 also binds to mRNAs encoding energy metabolism effectors in human cells, and *Igf2bp2* suppression in visceral or subcutaneous fat correlates with insulin resistance in diabetic patients.

Remarkably, Igf2bp2 binds >55% of cellular lncRAP2, but not as a client RNA. Instead, lncRAP2 and Igf2bp2 interact to program white adipocyte development and metabolism. Supporting this conclusion, we show that depleting either lncRAP2 or Igf2bp2 in mature adipocytes causes tightly corresponding changes in both transcriptome and proteome. mRNAs encoding energy metabolism effectors are selectively destabilized, in both cases limiting lipid breakdown. We thus expect that disrupting lncRAP2-Igf2bp2 complexes in mature adipose tissue will cause fat accumulation, increasing obesity, and thereby propensity for diabetes.

Mechanistically, changes in protein levels of Igf2bp2 client mRNAs mirror changes in their mRNA abundance, indicating that neither lncRAP2 nor Igf2bp2 directly affects mRNA translation. lncRAP2-Igf2bp2 complexes thus fine-tune energy metabolism primarily by modulating client mRNA stability. Although inhibiting Igf2bp2 phenocopies the molecular and physiological effects of lncRAP2 inhibition, both interact with additional proteins, such that these effects must reflect perturbation of many functions in addition to those exerted in partnership with each other.

Our characterization of a lncRAP2-Igf2bp2 interaction as a posttranscriptional regulatory program in adipocyte metabolism echoes the finding that the Airn and HIF1A-AS2 lncRNAs are functional Igf2bp2 cofactors in the developing brain (Mineo et al., 2016) and in cardiomyocytes (Hosen et al., 2018), respectively. Igf2bp2 thus appears to bind distinct lncRNAs to regulate distinct targets in diverse tissues. Given that lncRAP2 is adipose-specific and conserved in mice and in humans, it represents an attractive target to selectively modulate Igf2bp2 activity within fat tissue to treat or prevent the progression of obesity-linked diabetes.

### Limitations of the study

The experimental and computational systems used in this study have limitations to consider. In our experiments designed to investigate the functional relationship between lncRAP2 and Igf2bp2, we did not consider whether lncRAP2 modulates the affinity of Igf2bp2 for client mRNAs. Although we identify a human lncRAP2 homolog and characterize its regulation, its specific functions were not tested. We demonstrate that polymorphisms genetically and structurally associated with human lncRAP2 are linked to metabolic diseases, but their impact on the expression or function of human lncRAP2/Igf2bp2 and their targets was not investigated.

## STAR★Methods

### Key resources table

**Table:** 

REAGENT or RESOURCE	SOURCE		IDENTIFIER
**Antibodies**			

anti-IGF2BP2	Abcam		Ab151463
anti-DDX47	Abcam		Ab128204
anti-Exosc6	Abcam		Ab50910
anti-Serbp1	Abcam		Ab57285
anti-HNRNPU	Abcam		Ab20666

**Bacterial and virus strains**			

One Shot^TM^ TOP10 *E*.*coli*	ThermoFisher		C404010

**Biological samples**			

Total RNA from Subcutaneous Adipose Tissue, BMI <24.99	Zen-Bio		RNA-T10-1
Total RNA from Subcutaneous Adipose Tissue, BMI 25.0–29.99	Zen-Bio		RNA-T10-2
Total RNA from Subcutaneous Adipose Tissue, BMI >30.0	Zen-Bio		RNA-T10-3
Human MTC^TM^ Panel I	Takara		636742
Human MTC^TM^ Panel II	Takara		636743
Human Testis Total RNA	Takara		636533
Human Adipose Tissue Total RNA	Takara		636558
Mouse Total RNA Master Panel	Takara		636644

**Chemicals, peptides, and recombinant proteins**			

Paraformaldehyd	Electron Microscopy Sciences		15713
TRIzol Reagent	Thermo Fisher Scientific		15596026
Oil-red O	Sigma-Aldrich		O1391
Glutaraldehyde (25%)	Electron Microscopy Sciences		16216
Glycine	Sigma-Aldrich		G7126-10MG

**Critical commercial assays**			

Lipofectamine RNAiMAX	Invitrogen		LMRNA015
Adipolysis Assay Kit	Cayman Chemical		10009381
miRNAeasy Kit	Qiagen		n/a
Superscript II reverse transcriptase	Invitrogen		18064-022
Fast SYBRTM Green Mastermix	ThermoFisher		4385610
DC Protein Assay Kit II	Bio-Rad		5000112
ECL Plus Western Lightning Reagent	Perkin Elmer		NEL102
FirstChoice RLM-Race Kit	Invitrogen		AM1700
Gel Extraction Kit	Qiagen		n/a
TopoTA Cloning	Thermo Fisher Scientific		K4575J10
PARIS Kit cell fractionation	Life Technologies		AM1921

**Deposited data**			

RNA- and RIA-seq are deposited at GEO			GSE190047
Proteomics raw data at MassIVE			MSV000088559

**Experimental models: Cell lines**			

3T3-L1	ATCC		ATCC-CL-173

**Experimental models: Organisms/strains**			

C57BL/6J Wild-type mice, male and female mice, age 4–6 weeks	Jackson Laboratory		000664

**Oligonucleotides**			

All Oligonucleotides are listed in Table S1	N/A	N/A	

**Software and algorithms**			

Bowtie2	Langmead and Salzberg (2012)		http://bowtie-bio.sourceforge.net/bowtie2/index.shtml
DESeq	Anders and Huber (2010)		N/A
MACS2	Zhang et al. (2008)		N/A
Samtools	(Li et al., 2009)		http://samtools.sourceforge.net/
Fastqc	Andrews, 2010		https://www.bioinformatics.babraham.ac.uk/projects/fastqc/
Fastx_clipper, fastx_trimmer	Hannon et al., 2010		http://hannonlab.cshl.edu/fastx_toolkit/index.html
STAR v2.6.1	Dobin et al. (2013)		https://github.com/alexdobin/STAR
ImageJ	(Schneider et al., 2012)		https://imagej.nih.gov/ij/
BEDTools	Quinlan and Hall (2010)		N/A

**Other**			

heat inactivated newborn calf serum (HI-NCBS)	Life Technologies		26010-074
heat inactivated fetal calf serum (FBS)	Sigma-Aldrich		F2442
Collagenase A	Roche		10103578001
BSA	Sigma-Aldrich		A7906
Insulin	Sigma-Aldrich		11882
Dexamethasone	Sigma-Aldrich		D4902
3-isobutyl-1-methylxanthine (IBMX)	Sigma-Aldrich		I5879
Rosiglitazone	Cayman Chemical		71742
3,3,5-triiodo-L-thyronine (T3)	VWR		100567-778
Indomethacin	Sigma-Aldrich		I7378
Isoproterenol	Sigma-Aldrich		I6504
HALT protease and phosphatase Inhibitor	Thermo Fisher Scientific		78442
Protein A/G beads	Santa Cruz		sc-2003
Biotin-XX, SSE	Thermo Fisher Scientifc		B1606
SUPERRaseIn RNase Inhibitor	Life Technologies		AM2694
MyOne Streptavidin C1 magnetic beads	Life Technologies		65001

### Resource availability

#### Lead contact

Further information and requests for resources and reagents should be directed to the lead contact, Marko Knoll (markoknoll@gmail.com).

#### Materials availability

This study did not generate new unique reagents.

### Experimental model and subject details

#### Mice

C57BL/6J mice were bred in house or purchased from Jackson Laboratories (stock # 000664). All mice were housed under a 12 h light/dark cycle at constant temperature (20°C). All procedures were performed according to protocols approved by the Committee on Animal Care at the Massachusetts Institute of Technology.

### Method details

#### Isolation of primary cells and tissues

6–8 male 2–4 week old mice were sacrificed by CO_2_ asphyxiation and interscapular brown adipose tissue (BAT) and subcutaneous (inguinal) white adipose tissue (scWAT) was harvested into room temperature plain DMEM (Sigma, # 56499C). The fat pads were transferred into a well of a 6 well plate and minced with scissors for 5 min. Minced tissues were then transferred into a 50 mL conical tube with 3 mL Hank's balanced salt solution (Gibco, # 14175-095) supplemented with 0.2% collagenase A (Roche, # 10103578001) and 2% BSA (Sigma-Aldrich, # A7906) using a 1 mL pipet tip with the tip cut off to allow aspiration of larger pieces. The tissues were incubated agitating (350rpm) and repeated vortexing every 5 min for 10 s at 37°C for 30 min or 20 min for scWAT. Following collagenase digestion, 10 mL room temperature plain DMEM was added and cells were filtered through a 70 μm mesh filter (Corning, # 352350). Mature adipocytes and the stromal vascular fraction (SVF) were separated by centrifugation at 700 g for 5 min. The supernatant was removed and the SVF resuspended in 10 mL room temperature plain DMEM followed by additional filtering through a 30 μm mesh filter (Miltenyi Biotec # 130-041-407) and subsequent centrifugation at 700 g for 5 min. The SVF from subcutaneous white fat pads (scWAT) of 8 mice were then resuspended in 10 mL DMEM supplemented with 10% heat inactivated new born calf serum (HI NCBS, LifeTechnologies/Gibco, # 26010-074) and plated on two 10 cm dishes (Corning, # 430293). The SVF from interscapular brown fat pads of 6–8 mice were then resuspended in 6 mL DMEM supplemented with 10% HI NCBS and plated on 3 wells of a six well plate (Corning, #3506). After 4 and 24 h, the medium was replaced by fresh, pre-warmed DMEM/10% HI NBCS at 37°C and with 5% CO_2_. Cells were grown to confluence and then passaged no more than two times before seeding the pre-adipocytes for differentiation.

#### Cell culture

Pre-adipocytes derived from BAT were cultured to confluence and then subsequently overgrown for 4–6 additional days until growth arrested. The cells were then induced to differentiate by culturing them for two days in induction medium consisting of DMEM supplemented with 10% fetal bovine serum (FBS, Sigma-Aldrich F2442) and 850 nM insulin (Sigma-Aldrich #I1882), 0.5 μM dexamethasone (Sigma-Aldrich #D4902), 250 μM 3-isobutyl-1-methylxanthine (IBMX, Sigma-Aldrich #I5879), 1 μM rosiglitazone (Cayman Chemical # 71742), 1 nM 3,3,5-triiodo-L-thyronine (T3, VWR # 100567-778) and 125 nM indomethacin (Sigma-Aldrich #I7378). Subsequently, the induction medium was replaced with DMEM supplemented with 10% FBS and 160 nM insulin and 1 nM T3 for another two days. The cells were then cultured in DMEM 10% FBS and 1 nM T3 until day 8 of differentiation, and the medium was replaced every other day. Pre-adipocytes from scWAT were cultured similar but the induction medium and following medium did not contain T3.

#### Cell stimulation

Mature adipocyte cell layers were washed twice in plain pre-warmed DMEM and stimulated with 1 μM isoproterenol (Sigma-Aldrich I6504). After 6 h of stimulation, the cells were washed once with cold PBS and RNA was harvested using TRizol or QIAzol lysis reagent as described below. For immunoblotting, the cultures were harvested after stimulation with isoproterenol after washing with cold PBS on ice and adding 40 μL RIPA buffer per well of a 6 well plate or 100 μL RIPA buffer to a 10 cm dish.

#### siRNA transfection

Was performed as described (Isidor et al., 2016). Briefly, on day 4 of differentiation, adipocytes were transfected with 5 nM siRNA (Sigma-Aldrich) using 5 μL/mL Lipofectamine RNAiMAX diluted in Opti-MEM I Reduced Serum Medium (Life Technologies). The cultures were analyzed on day 6 of differentiation.

#### Glycerol release

Following addition of fresh medium, cells were stimulated with isoproterenol. Cell culture medium was collected after 24h of stimulation and stored at−20°C. Glycerol release was measured using the Adipolysis Assay Kit (Cayman Chemical, 10009381) following the instructions of the manufacturer.

#### Quantitative PCR

Total RNA was isolated from tissues or cells using TRizol or QIazol reagent (LifeTechnologies/Ambion) and a miRNAeasy kit (Qiagen). 300 ng were reverse transcribed using Superscript II reverse transcriptase (LifeTechnologies/Invitrogen) using random hexamers (LifeTechnologies/Invitrogen). The cDNA was diluted 1:10 and 2.5 μL for a 96 well plate or 1 μL for a 384 well plate were used for quantitative Real-time PCR. qPCR was carried out on an ABI7900HT Fast real-time PCR system (Applied Biosystems) and analyzed using the delta delta Ct method normalized to 18S if not stated otherwise. Results are shown as pooled data from 3–4 independent experiments displaying the average and SEM.

#### RNA sequencing analysis

Poly A+ RNA sequencing (TrueSeqStrandedPolyA) was performed on RNA samples using a Nextseq genome sequencer (Illumina). RNA-seq paired-end reads were aligned to the mouse genome (mm9; NCBI Build 37) using STAR v2.6.1 (Dobin et al., 2013) provided with a splice junctions databse (NCBIM37.67) and with default parameters and “--sjdbScore 2".mRNA sequencing was performed on three independent experiments. Differential analysis and counts per millions (cpm) were obtained using DESeq (Anders and Huber, 2010) as described (Alvarez-Dominguez et al., 2017a).

#### Immunoblotting

Lysates were centrifuged for 10 min at 13,000 g to remove debris, and NuPAGE sample buffer and reducing buffer (LifeTechnologies) were added after measuring and adjusting the samples for protein concentration (DC protein assay kit II, Bio-Rad). 2–20 μg protein per sample were separated for 2–4 h at 60–100 V using 8% 26 well NuPAGE Bis-Tris Midi gels in MOPS or MES buffer (LifeTechnologies) and in Criterion cells (Bio-Rad) using respective adapters (LifeTechnologies). Protein was wet transferred to polyvinylidene fluoride (PVDF) membranes (Immobilon P, Millipore) in Criterion blotter cells (Bio-Rad) using 2 x NuPAGE Transfer buffer (LifeTechnologies) with 10% methanol for 25 min at 1 A. After blocking the membrane in filtered (Nalgene # 595-4520) TBS-T (50 mM Trisbase, 150 mM NaCl, 0.1% Tween 20 [Sigma-Aldrich #P1379]) with 3% BSA (BSA, Sigma-Aldrich # A7906) for 1 h, blots were incubated in primary antibody diluted in TBS-T 3% BSA sealed in hybridization bags and gently shaking at 4°C overnight, then washed three times for 10 min in TBS-T, incubated in secondary antibody (Cell Signaling Technologies) diluted in TBS-T 3% BSA gently shaking for one hour at room temperature, and then washed again three times for 10 min in TBS-T. Antibody binding was visualized using ECL Plus Western Lightning reagent (PerkinElmer NEL 102) and blots were exposed to film (Kodak BioMax MR Film, Carestream Health Inc # 8701302). Films were scanned without adjustments using an Epson scanner. All immunoblotting data shown were reproduced with almost identical results in at least one and typically two to three additional independent experiments.

#### Oil-red O staining of brown adipocyte cultures

Cells were washed in PBS and fixed in 3.7% formaldehyde solution for 1 h, followed by staining with Oil Red O for 1 h. Oil Red O was prepared by diluting a stock solution (0.5 g of Oil Red O (Sigma) in 100 mL of isopropanol) with water (6:4) followed by filtration. After staining, plates were washed twice in water and photographed.

#### 5′ and -3′ RACE

The 5′ and-3′ ends of lncRAP2 was determined using the FirstChoice RLM-Race Kit from Ambion following the manufacturers instructions. Primers were designed accordingly and can be found in Data S3. Resulting gel bands were excised from the gel and purified using the Gel Extraction kit (Qiagen), cloned into a TopoTA vector (Thermo Fisher Scientific) and sequenced using the M13 fwd and rev primers supplied by the kit.

#### Cell fractionation

To separate the nuclear from the cytoplasmic fraction differentiated 3T3-L1 adipocytes were harvested and 1 million cells were used to isolated the fractions using the PARIS kit (Life technologies) according to the manufacturers instructions. Separated fractions were then analyzed using Real-time PCR and gene specific primers.

#### Single molecule FISH

Single molecule RNA FISH and fluorescence microscopy were described previously (Alvarez-Dominguez et al., 2014). Briefly, antisense probes were designed to span the exons of lncRAP2 (Data S3) and coupled to Cy5. Probes were hybridized at 2ng/μl final concentration. The maximum projection of FISH image z-stacks in the DAPI channel was merged with the z-slice of maximum contrast in the DIC channel, and the composite was used to identify cells. Images in the Cy5 channel were compared to those in the GFP control channel to detect diffraction-limited spots representing RNA transcripts using fixed pixel intensity thresholds. For image presentation, enhanced contrast in the DAPI channel was used to emphasize nuclear counterstaining boundaries.

#### Ribo-seq analysis

Ribosome profiling sequencing reads were clipped to remove 3′ linkers using fastx_clipper from the FASTX-Toolkit (http://hannonlab.cshl.edu/fastx_toolkit/index.html), discarding non-clipped reads or reads <25nt after linker clipping (“-l 25 -c” parameters), and

we usedfastx_trimmer to remove the first 5′ nucleotide from each read, as it often reflects an untemplated addition during cDNA generation. Pre-processed reads were then aligned to the mouse genome (mm9; NCBI Build 37) using STAR v2.6.1 with default parameters and “--seedSearchStartLmax 20 --sjdbOverhang 39 --outSJfilterOverhangMin 30 8 8 8 --sjdbScore 2”. To measure gene-level and region-level (CDS, intron, 5′UTR, and 3′UTR) expression from uniquely-aligned reads, we quantified read counts using HTseq-count. Only genes for which a read count could be obtained in each sample and replicate were retained, and counts were then normalized as counts per million mapped reads (cpm) using only reads from these genes.

#### Translation efficiency analysis

Gene-level translation efficiency was calculated as the ratio of normalized Ribo-seq read density (cpm) to the normalized RNA-seq read density (cpm).

#### Ribosome release analysis

We computed the ribosome release score using the RRS program (Guttman et al., 2013) (default parameters and “–n false”“-f false”), which measures the expected ribosome release after encountering a stop codon by calculating the ratio of the number of Ribo-seq reads in the ORF to the number of Ribo-seq reads in the 3′UTR, and dividing it by the same ratio calculated for RNA-seq reads (Guttman et al., 2013). For mRNAs, we used the longest annotated CDS and its 3′UTR to calculate the RRS. For lncRNAs, 5′ and -3′′UTRs, the RRS was computed for ORFs in all three possible frames whose 3′UTR was defined as the region between the stop codon and the beginning of the next ORF (in any frame) using the FindORFs utility (Guttman et al., 2013) (default parameters and “–c false -u true”). Only transcripts with a non-zero RRS were considered for all analyses.

#### RNACoimmunoprecipitation (RIP)

RNA coimmunprecipitation was done as described (Rinn et al., 2007). Mature adipocytes were harvested using trypsin. 1 × 10^7^ re-suspended in 2mL 1X PBS, then lysed in nuclear isolation buffer (2mL nuclear isolation buffer + 6mL water, premixed) for 20 min. Nuclei were pelleted by centrifugation at 2,500 g for 15 min. Supernatant was discarded and nuclei were re-supended in 1mL RIP buffer containing the HALT protease and phosphatase inhibitor (Thermo scientific) and split into two fractions and mechanically sheared using a Dounce homogenizer with 20 strokes. Nuclear membrane and debris were pelleted by centrifugation at 13,000 rpm for 10 min at 4°C. The supernatant was pre-cleared by adding 30μL slurry of protein A/G beads (Santa Cruz, sc-2003) and incubation for 2 h at 4°C on a rotator. Beads were removed by centrifugation at 2500 g for 1 min and 10% of the supernatant was removed to a new tube (10% input) and the rest was incubated with antibodies to Suz12 (6μg; abcam, ab12073), IgG (10μg; abcam, ab37415), Ezh2 (8μg; abcam, ab3748), hnRNPU (8μg; abcam, ab20666), CoREST (8μg, santa cruz, sc23449) or no antibody for 3h at 4°C on a rotator. Then 60μL slurry of protein A/G beads were added for 2h at 4°C on a rotator. Beads were pelleted by centrifugation at 2500 rpm for 30s and washed 3 times in 500μL RIP for 10 min each followed by one wash with 1X PBS. For the isolation of RNA, the beads were re-suspended in TRIzol after the last wash step and isolated according to the manufacturers instructions. The RNA pellet was re-suspended in 10μL dH_2_O and was directly used for reverse transcription using random hexamers and SuperScript II (Invitrogen). Analysis was done by real-time PCR and sequencing.

#### RNA interactome analysis followed by sequencing (RIA-seq)

RNA FISH probes were biotinylated using Biotin-XX, SSE (Thermo Fisher). As controls, we also used probes against H19 (Data S3) and Bloodlinc (Alvarez-Dominguez et al., 2017a). RIA-seq was conducted as described (Chu et al., 2011; Kretz et al., 2013). Briefly, 40 million day 6 differentiated adipocytes (3T3-L1) cells were harvested and crosslinked with 1% Glutaraldehyde for 10min at room temperature on a shaker, and the reaction was quenched with 0.125M glycine for 5min. Cells were collected by spinning at 2000g for 5min, resuspended in 4mL ice-cold PBS, and aliquoted to 4 collection tubes followed by resuspension in Lysis Buffer supplemented with Halt Protease and phosphatase inhibitor cocktail (Thermo Fisher, 100X) and with SUPERaseIn RNase Inhibitor (Life Technologies). Lysates were immediately sonicated in a Bioruptor (Diagenode) at 4°C using highest settings with 30 s ON and 45 s OFF for 3:45 h. The sonicated samples were spun at 16100g for 10min at 4°C, and the supernatant was then transferred to a new tube, with 10% taken as input RNA control (in Trizol). Hybridization Buffer and 100pmol of the probes were added to the supernatant and incubated at 37°C for 4h in a rotator. 100μL of pre-washed Dynabeads MyOne Streptavidin C1 magnetic beads (Life Technologies) were then added and incubated for 30 min at 37°C in a rotator, captured by magnets (Invitrogen), and washed with Wash Buffer for 5 min at 37°C for a total of five times. After the last wash, beads were resuspended in 1mL Wash Buffer and then lysed using TRIzol.

#### RIA-seq analysis

Raw reads (40bp unpaired and non-strand-specific) were mapped to mm9 using bowtie2 (Langmead and Salzberg, 2012). Peak calling was performed on uniquely-mapped reads relative to input control using MACS (Zhang et al., 2008) with default parameters and “--bw = 300--mfold 10 30”. High-confidence peaks were identified based on several criteria. First, peak coverage was quantified using BEDTools (Quinlan and Hall, 2010), and only peaks with an average per-base coverage greater than 2 reads were considered. Then peaks whose read density enrichment was at least 10-fold greater than the background model were considered enriched. Finally, peaks in the experimental sample overlapping peaks in the input control were excluded from further analysis.

#### Comprehensive identification of RNA-binding proteins by mass spectrometry (ChIRP-MS)

ChIRP-MS was performed as described (Chu et al., 2015) with the biotinylated probes used for RIA-seq. Since the first capture using even and odd probes didn't result in pull-downs, we used all 48 exon probes for pull-down and intron, Bloodlinc, H19 probes as well as no probe control and RNAse A treated samples as control. Briefly, 100 million day 6 differentiated adipocytes from 3T3-L1 cells were harvested and washed twice in PBS, crosslinked in 3% formaldehyde for 30min, then quenched with 0.125M glycine for 5min, and collected by spinning at 2000g for 5 min. Cells were lysed and sonicated for 2:30 h. Lysates were pre-cleared by incubating with 50μL washed magnetic beads at 37°C for 30min and retrieving them with magnets before proceeding to hybridization with probes overnight at 37°C in a rotator. The following day, beads were added and incubated for 30 min at 37°C in a rotator, then washed five times with Wash Buffer but not eluted as described in (Chu et al., 2015); instead, beads were boiled for 10min at 95°C in Laemmli Buffer, then beads and buffer were separated on a NuPAGE 4–12% Bis-Tris gel, followed by silver staining to identify differential bands. The whole gel lane was then excised, trypsinized, reduced, alkylated, and trypsinized at 37°C overnight. The resulting peptides were extracted, concentrated, and HPLC-purified. Effluents were analyzed using an Orbitrap Elite (ThermoFisher) mass spectrometer in nanospray configuration operated in data-dependent acquisition mode, where the 10 most abundant peptides detected using full scan mode with a resolution of 240,000 were subjected to daughter ion fragmentation in the linear ion trap. A running list of parent ions was tabulated to an exclusion list to increase the number of peptides analyzed throughout the chromatographic run.

#### ChIRP-MS analysis

Mass spectrometry fragmentation spectra were correlated against a custom peptide database, formed by downloading all RefSeq species-specific (mm9) entries, and against a database of common contaminants (keratin, trypsin, etc.) using Mascot (Matrix Science) 2.5.1 and the Sequest algorithm (Thermo). The resulting Mascot search results were uploaded into Scaffold (Proteome Software), and a minimum of two peptides and peptide threshold of 95% and protein threshold of 99% were used for identification of peptides and positive protein identifications. Proteins enriched in ChIRP-MS experiments were identified based on several criteria. First, only proteins identified by 2 or more unique peptides in the differentiated sample were considered. Then proteins whose total peptide count was at least 10-fold greater in the exon probes versus intron probes were considered enriched. Finally, proteins introduced during sample preparation and purification (e.g. streptavidin, albumin) were excluded from further analysis. To distinguish lncRAP2-specific interactors from proteins that generally bind RNA during its processing, we compared the proteins enriched by lncRAP2 with those enriched by Dleu2 (Alvarez-Dominguez et al., 2017a), Neat1 and Malat1 (West et al., 2014), and U1, U2, and Xist (Chu et al., 2015), which also filtered for identification by 2 or more unique peptides, for enrichment relative to their control sample greater than 3-fold, and for known contaminants. Proteins enriched by each RNA were ranked according to their total peptide counts, and rankings were compared.

#### Silver staining

After proteins were separated on an Acryl-amid gel (see Western blot), the gel was fixed in 40% EtOH with 10% HAc for 1 h then washed 2 × 20 min in 30% EtOH and once for 20 min in water. The gel was sensitized for 1 min in 0.02% Na2S2O3 following 3 × 20 s wash in water and 20 min incubation in cold (4°C) 0.1% AgNO3 solution. The gel was washed 3 × 20 s in water and developed in a new chamber in 3% Na2CO3 with 0.05% formaldehyde. As soon as bands became visible the reaction was stopped by a short wash in water and then incubation in 5% HAc. All lanes were cut out and processed (see ChIRP-MS analysis).

#### Gene set and pathway enrichment analysis

Gene lists were analyzed for enrichment of genes grouped by biological process ontology or by curated annotations from the Molecular signatures database with GSEA (Subramanian et al., 2005) using default parameters and “-metric log2_Ratio_of_Classes”.

#### Motif enrichment analysis

Genomic sequences from regions of interest (e.g. UTRs) were searched for matches to a database of TF recognition sites (Saint-Andre et al., 2016) for TFs expressed in the relevant cell type using FIMO (Grant et al., 2011) as described in (Alvarez-Dominguez et al., 2017b) with minor modifications: a Markov model of sequence nucleotide composition was used as the background model for motif matching (to normalize for biased distribution of individual letters in the examined sequences), and motifs with an odds ratio>2 and q-value<0.05 (Fisher's exact test) relative to 10 randomly-shuffled controls were considered significantly enriched.

#### Chromatin interaction analysis

Processed ChIA-PET, Hi-C, and capture Hi-C datasets were downloaded and visualized in triangle heatmap mode using the UCSC genome browser or in arc mode using the Washington University Epigenome browser, with default normalization and resolution settings and fixed display values.

#### Additional bioinformatics methods

All sequencing reads were quality-checked with FastQC (http://www.bioinformatics.babraham.ac.uk/projects/fastqc/). Genome-wide read density maps were generated by MACS2 using the “--bdg" option, normalized by RSeQC (Wang et al., 2012) using the “normalize_bigwig.py” function, and visualized using BEDTools and the UCSC genome browser. Signal coverage and signal change surrounding regions of interest (e.g. DMRs, enhancer sites) were visualized using the ngs.plot R package (Shen et al., 2014). Data heatmaps were generated using the heatmap.2 function of the gplots R package (http://CRAN.R-project.org/package=gplots).

### Quantification and statistical analysis

No statistical methods were used to predetermine sample size or remove outliers. The statistical difference between two sets of paired count data (e.g. motif matches in test vs. randomly-shuffled sequences) was assessed by a Fisher's exact test using the fisher.test R implementation with default parameters. For unpaired data, a Shapiro-Wilk normality test was first performed using the shapiro.test R implementation with default parameters; for normally distributed data we then used a two-sided ttest (t.test R implementation with default parameters) to assess confidence on the measured difference of their mean values. For unpaired data that don't follow a normal distribution, we used a non-parametric Wilcoxon rank-sum test to determine if they belong to the same distribution. Variance was represented as mean ± SEM of n = 3 replicates unless otherwise specified.

### Additional resources

#### Human lncRAP2 sequence

>hg19_chr10: 94,176,182-94,180,397

ATCCGTTACCCAACGGCTGTCGAAAGAGGAGCACGACGTGAACTTCCACCGACAATCGTTTAAATACTGCAGGCGAAGGACGGGTTCTTATT

GTTCCTTCAGGCAAACCGGCAGCTGAGTCCAGCTTGTGAACTTGAACTTG

AACTTGCTGAAGAAGCTCCCGGCGGCCCTCTGCTGGCCGCCGCCTTTGCC

AGAGGGAGAGGCTGGAGTCACCCTGTTGGAACCCATTTCTGGGGCTGGCA

CCTGTTGGGCTGCCCGGCCGCGCGTACCTGGTCCCATCGGGGGCTCTGCC

CACTCCGCTGATGACGCGGGTAGAAGGGAGGCCGCAGGGACACTCTGGGG

GGACTGTGCCGGGCGGGCACCCCCCAGCTGCTCACTGTGGGGTGCGGCAC

CGAGGCCTGGTTGGGCTGCAAGGAGACCGACTGGGATTCCCGGGCTGGTG

GCCGGGGAGACGGGGTAGAGGTGAGAAGCAAGAGCTCAGGAGGCCTCAGG

CCCCAGCGCTGTGGGGCTGCCGTTGTCGTTCTGGGTGGAGGTCTGGCCAA

ACCGGCTTTTGCCCCGAGTGAGGAATTCCTGCTCATTTTGGTGTTAGTGG

AGAGGTCGCTGTTCACAGCGGGGGTGGGGTTCGTCCCCTCCAGGCTAGTA

GGGAGCTGGCTGGTGCATTGCTGTGTTGCTGCCCTTCTGCCCTGTCTCCT

GATCCTGTTCAGACTCTGGGTGGCTCCTTTGAGTCTTCTCAGGCCGTGAA

AGATGGGAAAGGCAGGTTGTTGAAACCACTGCTGCTCCGGGCTGGCAGAG

ACCCTGAGCTGTACCTCCTGGGATGAAAGGAGAACCCAGGAGCAGGCAGG

TGACCACTGCTTCCTCATACTCCTCAGCAAGTCCTGGATCTCTTCCTGTC

TGTAACCCACGGATACCATGAACTTAGTTTGCAGGGGGTCCTCCCAGTGG

GAGAGTGGCTGCACACAGCGCTTAAGTTCCTCACCTTCATGACTCGCATT

CATCCATGGACCCCTCACAGTGTGCTCTGAAGGGCCTCTCTTCCTGCAAT

TGACAATGAGAAATTTCTTCAGCAAGTTTTCACTCTCCGGGGACATGGGG

GAGGGAATGGAATACATCCTGCTCAGTACCTGCTCCTATAGCTCCTTGAA

GTTCTGTCCATCGAAAAATAAGGATCCATTTACCAGTATATAGAGGATAA

CTCGCAGACGCCATACATCCACCAGCTCATATTTGTGGCCCTAGAAGAAT

TCCAGGGCAGCCTAAGGGGACTGCCACAAAAGGTATCCAGCTTGTTGCGA

AAGGCGAATTTATTCCTGAACCCAATCTGTGATGTTCATGTTAACATGGA

CAATACATTTCTGGTGACAATACACAATATCTGTGGACAATGCACTTCTG

GTAACAGCTTGCATAGCAGACACTTTTGGTGGAATTTGCTTCAGGCCTCT

TTCCTGCTGCTGTGAGCAGGTATTCACAAACTTCTCGCTAGCGTGCTTTG

TGATGAGGCAGAGATTCCTCACTATCGATCACAGCAAATACTTCACTATG

TTGGGGTGATCTGAGCCTTCATGATTCCTACTTTGTGGATGGTGTCTGGA

GGCTGGAGGAGATCTGCTGAGTCTCATCAATGAACTTCACGGCGGCTGCT

TTCCCAGTCAGGATGTCCCAGGCCAACCCCACCTTAGCCAGGTTGTCCTT

GACGATGGTCTCAAGGATCCTCGCTGCCAATAAGGATCCCTCCTCAGCAG

AGATGGCTGAGAGGCCCTGCGGCGTGTGGGACTTACTACTGGGCTTGGAG

TCAAGGTGTCCCAGGTCGGCTTAATTTTGGGAAAAGCTGGTGAGAAGCTT

GGTCCAAATCACCTGAAAAGAAAATCACTTGCATAGTTTACCTGAAGAAC

GGATTTCTATAATGGAATCAAAACACAATACTGGACAAAAATCAAATAAG

CAAAAGCAACAAACTGAAAAGGAAATGCTGAGTAAAAAAGAACTTAAAAA

TTAGAAAAATAAGAGAAAAAAGAACAAGAAGAACCAGTAACCTCAGCCAA

GCCAGGGACTTACATACGTGCTTGGAATTCCAAAGCAGCAGTTCCTTATG

AGGAAGAACTAAGCCTAGTAACAAGGCTGAGGATAATCTATGTGGCTTTC

TCATGCTTTGGTCTCAAGAACTCTTTACTCTTAAAGAAAATATATTGAGG

ACCACAAAGAGGATTTTTTATTGATATGGGTTACAGTTATGAATATTTAC

CTTATTAGAAATTAAAACCTCTAGGATGCTTCAATGGCCTTTTCTAGTTT

GAAAAGATAACAGGCTGGGTGTGGTGGCTCACGCCTGTAATTCCAGCACT

TTGGGAGGCCGAGGTGGGCAAATCACCTGAGCTCGGGAGTTCGAGAAAAG

GTATAAAAATGTTTGGCTTTTAAAGAGCCCACAATATCTACACTTAAAAT

ATTTCATTTTTTTCTTTAAACTCTAAATGATTGGTTTCAAAATGATGCCA

CAACTTAGCTGGCATTATGATAGTGTATAAGTATGTTCTGTTGTGTACGA

CACAATGAGCTTCATTATTACCATCTGCGACACTGAGGGGAAGATAGCTT

TCATCATATTTTCTCATTTAAAGTTTTGCCCAGTTTCATTTGCATAGATT

CCCTTTTTCCATGAGCTGCTATGTCAGTCTCAGCATCTTTCAATGTAGAG

TTTGCAGCTATGAGTTGAGAAAGCACATTTTCTACTCTTTTTAAGTGAAT

AATCCACTGTGCCTGGTGTACCTCTCCTTCAGCATAGGATAGGGACATCC

AGGTACTGGACCCGTCACTGGCACCTCAGTGGGGAGAACCCAGATGCCCC

TACATGATGTTTAAAGATGCTTTATATACATAAAAGTGCACAAATCATCA

GCCCACAGCTTGGTGACTGTTCACATATTGAACTCATCTATTTATCTAGT

ATCCAGGTCAAGAAACAGCCATTACAGCCCCCCAAGATCCCACACCCCTT

TTCCAGTCACTTTCTCTGCAGTGATAACCACTCTTCTGTATTTTGACAGC

ATAGATTCATTTTGCTTATTTTTGAACTTTACATACATGGATTCATACAG

TATTGGATCCTTTGTGTCTGCTTCCTTTGCTTAATTTGTTTTTGTTTGTT

TGTTTGTTTGTTTGTTTTTCTTGAGACAGAGTCTTGCTCTTGTTGCCCAG

GCTGGAGTGCAATGGCACGATCTCAGATCACTGCAACCTCCACCTCCTGG

GTTCAAGCAATTCTCCTGCCTCAGCCTCCCAAGTAGCTGGGATTATAGGA

GCTTGCCACCATGCCTGGCTAATTTTTGTATTTTTAGTTGAGACGGGGTT

TCACCATGTTGGCCAGGCTGGTCTCGAACTCCTTACCTCATGTTCCGCCT

GCCTCAGCCTCCCAAAGTGCTGGAATTACAGGCATGAACAACCACGCCTG

GTCCTTTGCTCAATATTTTTGTGAGATCCATCCATATTGTTTATTATTCT

AAATGCTCATTGTGTGACTGTAACACAATTTGTTAATTTGTTTATTCATT

TTACTGTTACTGGGCAGTTGAGTAGTTCTCAGTTTTCAGATGCTATAGTG

CTGCCATAAACATTCTTGTTCAGGTTTTTGGGGGACATATATATGGCTTT

CTGTTGGATATATATAAATATATTAAGGGTGTGGCTGAACAACCATTTGA

CAGTTTATGCTAACAAGGTGACTCGTGGTAGGCCCCTTAGGCCAGGTGAT

ATCAGCCTGACCTCCAGAGAGTGGGGTGGGGGCTGGAGACTGAGTTCAAC

CACATGGACAATAAGTCTATCATGTAATGAAGCCCCAGTAAAAACTCTGG

ATGCTGAAGCTCAGGTGAGTGTCCCTGATTGGCAGTACTCTATATGTGTT

GTCTCACACATCCAAATCAGCAGGGTAATGCATTCTGAGGACCCCAGAGG

CTTCACATTTGGAACCCTCTCAGACTCTGCTCTATCAATCTCTTTCTTTG

GCTAATTTTGATCTCTATCCTTTCCCTGAAATAAACTGTAACTGTGAGTA

TAACAG.

## Data Availability

• RNA-seq and RIA-seq data have been deposited at GEO (GSE190047) and are publicly available as of the date of publication. Proteomic raw data are deposited at MassIVE (MSV000088559). Accession numbers are listed in the key resources table. Original Western blot images and microscopy data reported in this paper will be shared by the lead contact upon request. • This paper does not report original code. • Any additional information required to reanalyze the data reported in this paper is available from the lead contact upon request.

## Supplemental information

Document S1. Figures S1–S5 and Table S1

Data S1. High-confidence lncRAP2-associated proteins and their peptide counts, related to Figure 2

Data S2. Enriched Igf2bp2-associated RNAs and their enrichment values, related to Figure 3

Data S3. Oligos and antibodies used in this study, related to STAR Methods

Data S4. Datasets used in this study, related to STAR Methods
