# Supplementary material for: An adipose lncRAP2-Igf2bp2 complex enhances adipogenesis and energy expenditure by stabilizing target mRNAs
Source: iScience. 2021 Dec 25;25(1):103680. doi: 10.1016/j.isci.2021.103680 (PMC8749451; doi:10.1016/j.isci.2021.103680)
Supplement: Document S1. Figures S1–S5 and Table S1 [file mmc1.pdf]

**iScience, Volume *25***

## Supplemental information

# An adipose lncRAP2-Igf2bp2 complex enhances adipogenesis and energy expenditure by stabilizing target mRNAs

**Juan R. Alvarez-Dominguez, Sally Winther, Jacob B. Hansen, Harvey F. Lodish, and Marko Knoll**

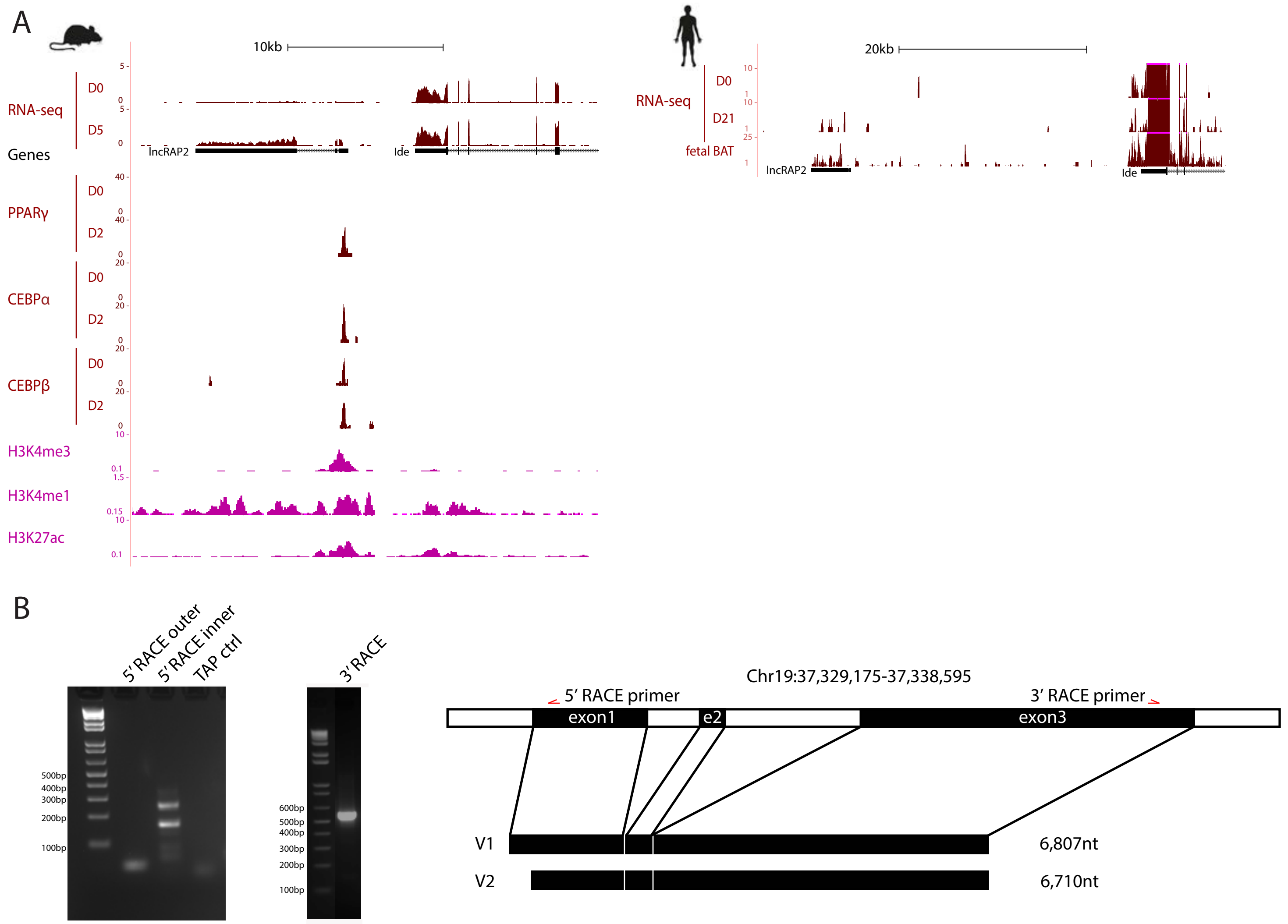

A
10kb
RNA-seq
D0
D5
Genes
lncRAP2
lde
PPARγ
D0
D2
CEBPα
D0
D2
CEBPβ
D0
D2
H3K4me3
H3K4me1
H3K27ac
20kb
RNA-seq
D0
D21
fetal BAT
lncRAP2
lde
B
5' RACE outer
5' RACE inner
TAP ctrl
500bp
400bp
300bp
200bp
100bp
3' RACE
600bp
500bp
400bp
300bp
200bp
100bp
Chr19:37,329,175-37,338,595
5' RACE primer
3' RACE primer
exon1
e2
exon3
V1
6,807nt
V2
6,710nt

**Figure S1. lncRAP2 is a conserved cytoplasmic RNA required for adipogenesis. Related to Figure 1.**

(**A**) lncRAP2 (transcription right-to-left) structure, expression, and regulation in brown adipocytes. Tracks show signal from sequencing studies of primary brown adipocytes from mouse (A) and human (Ding et al., 2018) (B).

(**B**) Rapid amplification of cDNA ends (RACE) delineates the lncRAP2 transcript. The genomic locus, spliced transcript variants (V1 and V2), RACE primers, and the amplified 5’ and 3’ end fragments sequenced are shown.

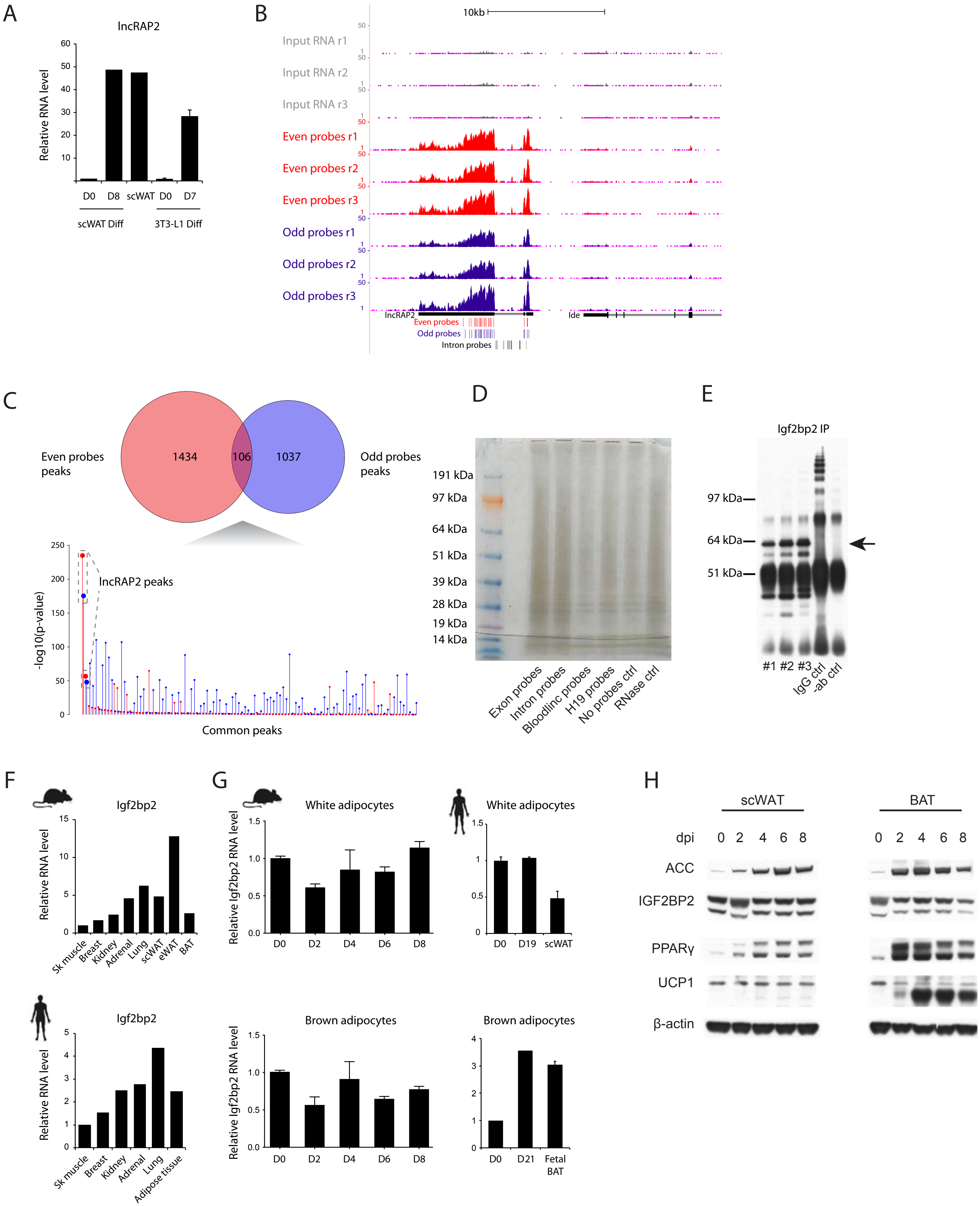
A
lncRAP2
Relative RNA level
D0
D8
scWAT
D0
D7
scWAT Diff
3T3-L1 Diff
B
10kb
Input RNA r1
Input RNA r2
Input RNA r3
Even probes r1
Even probes r2
Even probes r3
Odd probes r1
Odd probes r2
Odd probes r3
lncRAP2
Ide
Even probes
Odd probes
Intron probes
C
Even probes peaks
1434
106
1037
Odd probes peaks
lncRAP2 peaks
-log10(p-value)
Common peaks
D
191 kDa
97 kDa
64 kDa
51 kDa
39 kDa
28 kDa
19 kDa
14 kDa
Exon probes
Intron probes
Bloodlinc probes
H19 probes
No probes ctrl
RNase ctrl
E
Igf2bp2 IP
97 kDa
64 kDa
51 kDa
#1
#2
#3
IgG ctrl
-ab ctrl
F
Igf2bp2
Relative RNA level
Sk muscle
Breast
Kidney
Adrenal
Lung
scWAT
eWAT
BAT
Igf2bp2
Relative RNA level
Sk muscle
Breast
Kidney
Adrenal
Lung
Adipose tissue
G
White adipocytes
Relative Igf2bp2 RNA level
D0
D2
D4
D6
D8
White adipocytes
D0
D19
scWAT
Brown adipocytes
Relative Igf2bp2 RNA level
D0
D2
D4
D6
D8
Brown adipocytes
D0
D21
Fetal BAT
H
scWAT
BAT
dpi
0
2
4
6
8
ACC
IGF2BP2
PPARγ
UCP1
β-actin

**Figure S2. lncRAP2 forms a complex with proteins that regulate mRNA stability and translation. Related to Figure 2.**

(**A**) lncRAP2 is expressed at comparable levels in subcutaneous white adipose tissue (scWAT), upon *in vitro* differentiation of scWAT preadipocytes, and upon *in vitro* differentiation of 3T3-L1 preadipocytes.

(**B**) Specific and reproducible enrichment of spliced lncRAP2 by antisense purification. Tracks show signal from RNA interactome analysis by sequencing (RIA-seq) studies of differentiated white adipocytes. Locations of lncRAP2 exon- and intron-targeting antisense probe pools are shown below.

(**C**) lncRAP2 does not directly bind other RNAs. A minority of RIA-seq peaks are common to both lncRAP2 exon-targeting probe pools, and show poor enrichment or concordance between the probe pools, other than the peaks from capturing lncRAP2 itself.

(**D**) lncRAP2 antisense purification retrieves rich protein analytes compared to controls. The specific enrichment of protein analytes in differentiated 3T3-L1 adipocytes recovered by Silver stain of captured proteins before mass spectrometry analysis is shown.

(**E**) Specific native immunoprecipitation of Igf2bp2 (highlighted) in primary mouse white adipocytes. Western blot of captured proteins from an aliquot after pulldown with an Igf2bp2-specific antibody.

(**F**) *Igf2bp2* expression in select tissues. Relative expression in different organs and tissues from mouse (Alvarez-Dominguez et al., 2015) (top) and human (Uhlen et al., 2015) (bottom) is shown. Human adipose tissue pools samples from subcutaneous and visceral depots.

(**G**) *Igf2bp2* expression during an *in vitro* differentiation timecourse of mouse inguinal white adipose tissue and interscapular brown adipose tissue preadipocytes (left) and human subcutaneous adipose tissue and fetal interscapular brown adipose tissue preadipocytes (Ding et al., 2018) (right).

(**H**) Igf2bp2 protein levels during *in vitro* differentiation of mouse white or brown preadipocytes. Western blots for the indicated proteins are shown.

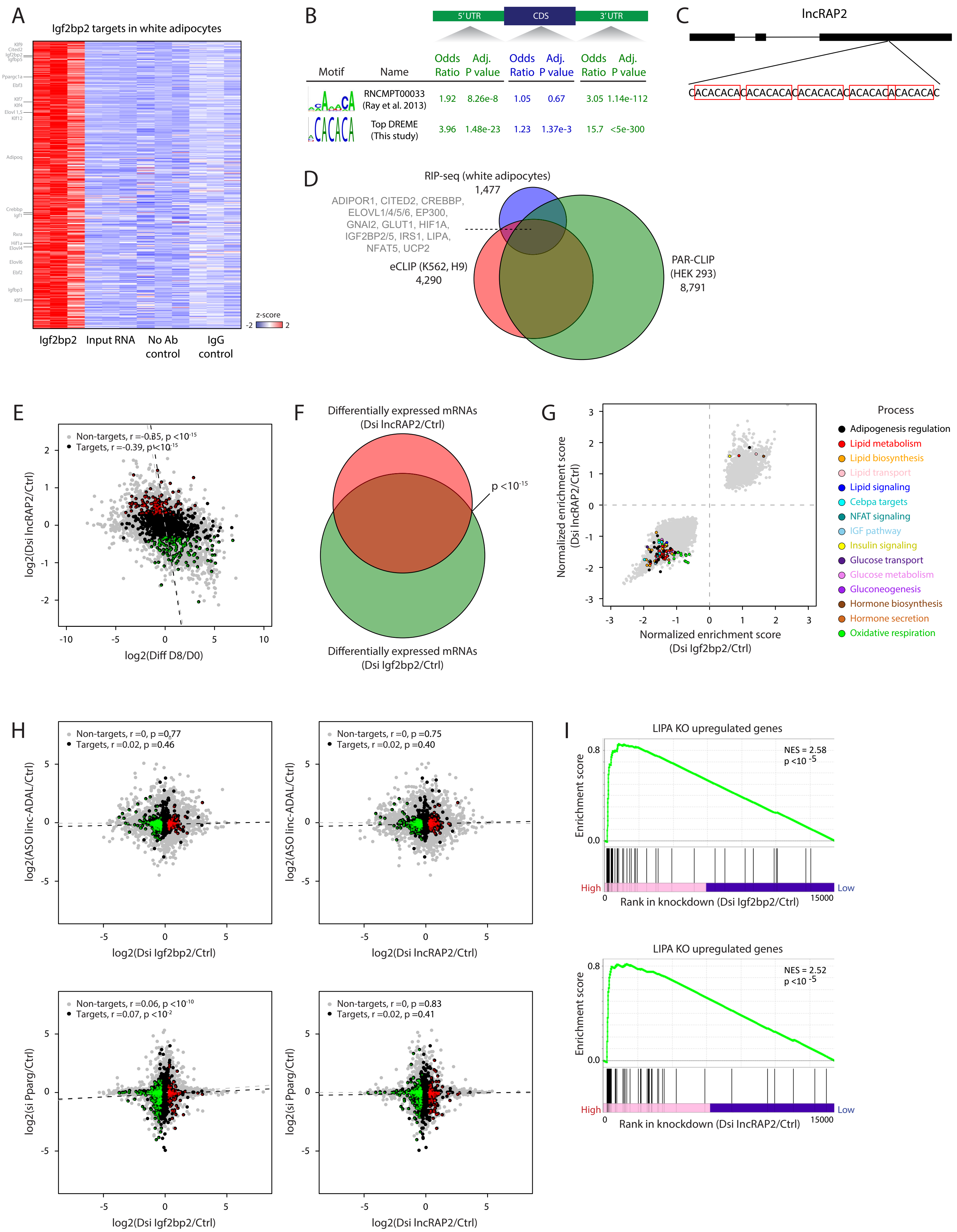

A
Igf2bp2 targets in white adipocytes
Klf9
Cited2
Igf2bp2
Igfbp5
Ppargc1a
Ebf3
Klf7
Klf4
Elovl 1,5
Klf12
Adipoq
Crebbp
Igf1
Rxra
Hif1a
Elovl4
Elovl6
Ebf2
Igfbp3
Klf3
z-score
-2
2
Igf2bp2
Input RNA
No Ab control
IgG control
B
5'UTR
CDS
3'UTR
Motif
Name
Odds Ratio
Adj. P value
Odds Ratio
Adj. P value
Odds Ratio
Adj. P value
RNCMPT00033 (Ray et al. 2013)
1.92
8.26e-8
1.05
0.67
3.05
1.14e-112
Top DREME (This study)
3.96
1.48e-23
1.23
1.37e-3
15.7
<5e-300
C
lncRAP2
CACACACACACACACACACACACACACACACACACACAC
D
RIP-seq (white adipocytes)
1,477
ADIPOR1, CITED2, CREBBP, ELOVL1/4/5/6, EP300, GNAI2, GLUT1, HIF1A, IGF2BP2/5, IRS1, LIPA, NFAT5, UCP2
eCLIP (K562, H9)
4,290
PAR-CLIP (HEK 293)
8,791
E
Non-targets, r =-0.35, p <10-15
Targets, r =-0.39, p <10-15
log2(Dsi lncRAP2/Ctrl)
log2(Diff D8/D0)
F
Differentially expressed mRNAs (Dsi lncRAP2/Ctrl)
p <10-15
Differentially expressed mRNAs (Dsi Igf2bp2/Ctrl)
G
Normalized enrichment score (Dsi lncRAP2/Ctrl)
Normalized enrichment score (Dsi Igf2bp2/Ctrl)
Process
Adipogenesis regulation
Lipid metabolism
Lipid biosynthesis
Lipid transport
Lipid signaling
Cebpa targets
NFAT signaling
IGF pathway
Insulin signaling
Glucose transport
Glucose metabolism
Gluconeogenesis
Hormone biosynthesis
Hormone secretion
Oxidative respiration
H
Non-targets, r =0, p =0.77
Targets, r =0.02, p =0.46
log2(ASO linc-ADAL/Ctrl)
log2(Dsi Igf2bp2/Ctrl)
Non-targets, r =0, p =0.75
Targets, r =0.02, p =0.40
log2(ASO linc-ADAL/Ctrl)
log2(Dsi lncRAP2/Ctrl)
Non-targets, r =0.06, p <10-10
Targets, r =0.07, p <10-2
log2(si Pparg/Ctrl)
log2(Dsi Igf2bp2/Ctrl)
Non-targets, r =0, p =0.83
Targets, r =0.02, p =0.41
log2(si Pparg/Ctrl)
log2(Dsi lncRAP2/Ctrl)
I
LIPA KO upregulated genes
NES = 2.58
p <10 -5
Enrichment score
High
Low
Rank in knockdown (Dsi Igf2bp2/Ctrl)
15000
LIPA KO upregulated genes
NES = 2.52
p <10 -5
Enrichment score
High
Low
Rank in knockdown (Dsi lncRAP2/Ctrl)
15000

**Figure S3. lncRAP2-Igf2bp2 target transcripts of metabolic effectors to potentiate energy expenditure. Related to Figure 3.**

(**A**) Igf2bp2 selectively binds mRNAs encoding adipogenic regulators and effectors. Enrichment of RNAs in native Igf2bp2 or control immunoprecipitates (n=3 replicates each) in mouse white adipocytes.

(**B**) Igf2bp2 targets enrich for the known Igf2bp2 binding motif predominantly within their 3'UTRs. Computational analysis of short motifs and their relative enrichment within the 5'UTR, coding sequence, and 3'UTR regions of transcripts bound by Igf2bp2 in mouse white adipocytes. The top DREME (Bailey, 2011) motif identified (bottom) and the experimentally inferred Igf2bp2 binding motif (Ray et al., 2013) (top) are compared.

(**C**) lncRAP2 harbors multiple Igf2bp2 binding motif instances. The sequence of five intact motifs (two are overlapping) and their location within the lncRAP2 transcript are shown.

(**D**) Most Igf2bp2 targets in mouse white adipocytes also copurify with Igf2bp2 in human cells. Overlap between 1,477 targets identified by RNA immunoprecipitation sequencing (RIP-seq) in mouse white adipocytes that have human homologs, those identified by enhanced crosslinking and immunoprecipitation (eCLIP) in human embryonic stem (H9) (Conway et al., 2016) and in erythroleukemia (K562) cells (Van Nostrand et al., 2016), and those identified by photoactivatable ribonucleoside-enhanced crosslinking and immunoprecipitation (PAR-CLIP) in human embryonic kidney (HEK 293) cells (Hafner et al., 2010). Common adipogenic regulator and effector targets identified by all three methods are highlighted.

(**E**) The induction/suppression of Igf2bp2 RNA targets with adipogenesis is reversed in lncRAP2-depleted cells. RNA changes for Igf2bp2 targets (black circles) and non-targets (gray circles) upon *in vitro* differentiation of lncRAP2-depleted vs. untreated mouse scWAT preadipocytes is shown. Genes significantly ($p < 0.05$) upregulated (red) or downregulated (green) upon lncRAP2 depletion are highlighted.

(**F**) Significant overlap ($p < 10^{-15}$, hypergeometric test) between mRNAs that are differentially expressed ($p < 0.05$, t-test) after lncRAP2 depletion (top) and after *Igf2bp2* depletion (bottom).

(**G**) lncRAP2/*Igf2bp2* depletion cause selective destabilization of transcripts encoding mediators of energy metabolism. Gene set enrichment analysis highlighting significant ($p < 0.05$) biological processes upon *Igf2bp2* vs. lncRAP2 depletion.

(**H**) No correlation between transcriptome-wide effects of depleting lncRAP2 or Igf2bp2 with those of depleting Pparγ or linc-ADAL (lincRNA for adipogenesis and lipogenesis). Shown are RNA changes for Igf2bp2/lncRAP2 targets (black circles) and non-targets (gray circles) upon antisense oligonucleotide (ASO)-mediated depletion of lnc-ADAL or siRNA-mediated depletion of Pparg in mature white adipocytes (Schupp et al., 2009; Zhang et al., 2018). Genes significantly ($p < 0.05$) upregulated (red) or downregulated (green) upon Igf2bp2 or lncRAP2 depletion are highlighted.

(**I**) Gene set enrichment analysis reveals significant overlap between the lncRAP2 and *Igf2bp2* depletion gene signatures and that of genes up-regulated (>=2 fold-change) at 6 months of age in lungs from lysosomal acid lipase gene knockout mice (Lian et al., 2005). NES, normalized enrichment score.

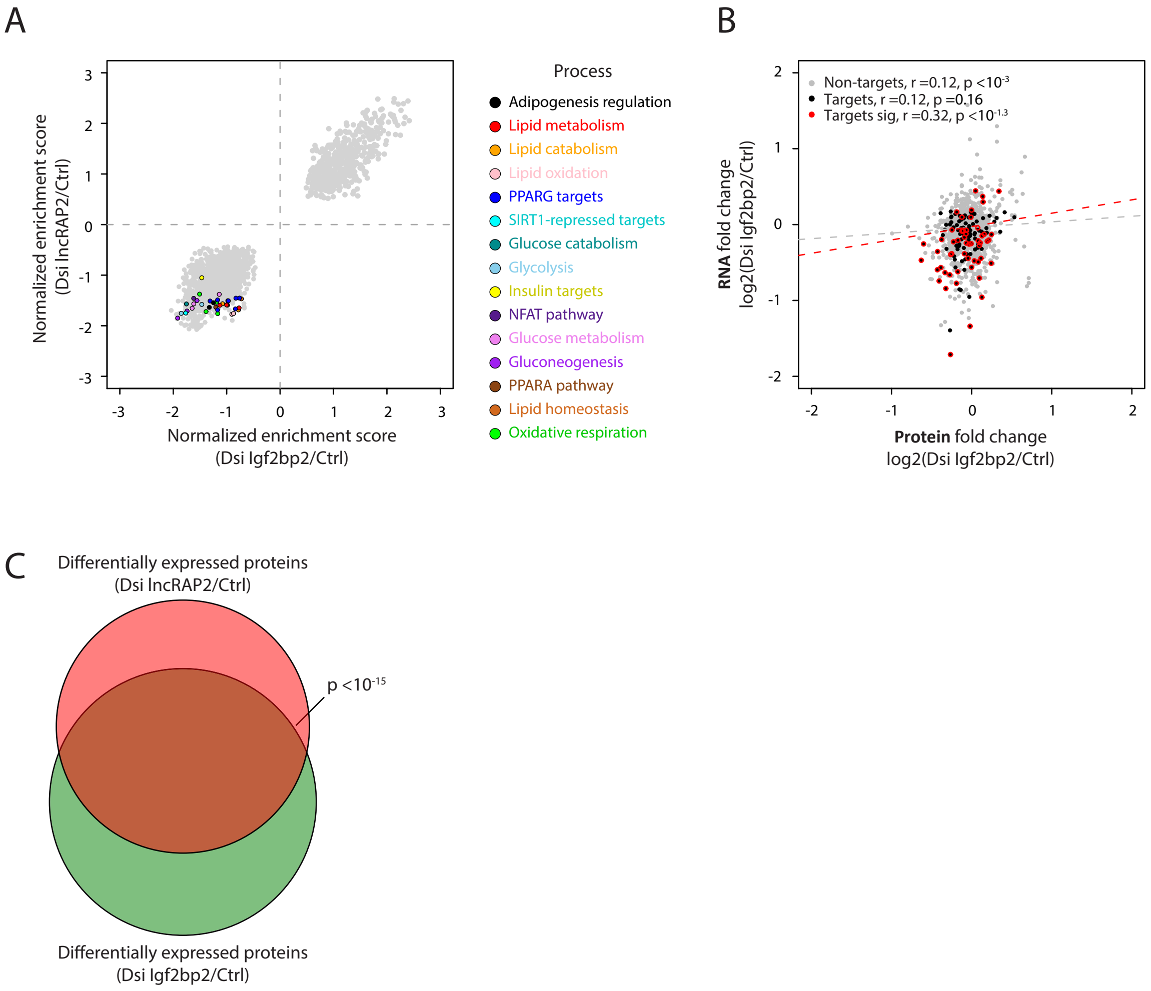
A
Normalized enrichment score (Dsi lncRAP2/Ctrl)
Normalized enrichment score (Dsi Igf2bp2/Ctrl)
-3 -2 -1 0 1 2 3
Process
Adipogenesis regulation
Lipid metabolism
Lipid catabolism
Lipid oxidation
PPARG targets
SIRT1-repressed targets
Glucose catabolism
Glycolysis
Insulin targets
NFAT pathway
Glucose metabolism
Gluconeogenesis
PPARA pathway
Lipid homeostasis
Oxidative respiration
B
Non-targets, r =0.12, p <10-3
Targets, r =0.12, p =0.16
Targets sig, r =0.32, p <10-1.3
RNA fold change log2(Dsi Igf2bp2/Ctrl)
Protein fold change log2(Dsi Igf2bp2/Ctrl)
-2 -1 0 1 2
C
Differentially expressed proteins (Dsi lncRAP2/Ctrl)
p <10-15
Differentially expressed proteins (Dsi Igf2bp2/Ctrl)

**Figure S4. lncRAP2-Igf2bp2 predominantly tune the levels of target mRNAs. Related to Figure 3.**

(**A**) lncRAP2 and *Igf2bp2* depletion cause coordinate reduction in levels of key energy metabolism effector proteins. Gene set enrichment analysis highlighting significant ($p < 0.05$) biological processes upon *Igf2bp2* vs. lncRAP2 depletion in mature white adipocytes.

(**B**) Coordinate RNA and protein changes upon *Igf2bp2* depletion. Global protein vs. RNA changes upon *Igf2bp2* depletion in mature white adipocytes, highlighting Igf2bp2 targets with protein changes that are significant after Igf2bp2 depletion ($p < 0.05$, red) or not (black).

(**C**) Significant overlap ($p < 10^{-15}$, hypergeometric test) between proteins that are differentially expressed ($p < 0.05$, t-test) after lncRAP2 depletion (top) and after *Igf2bp2* depletion (bottom).

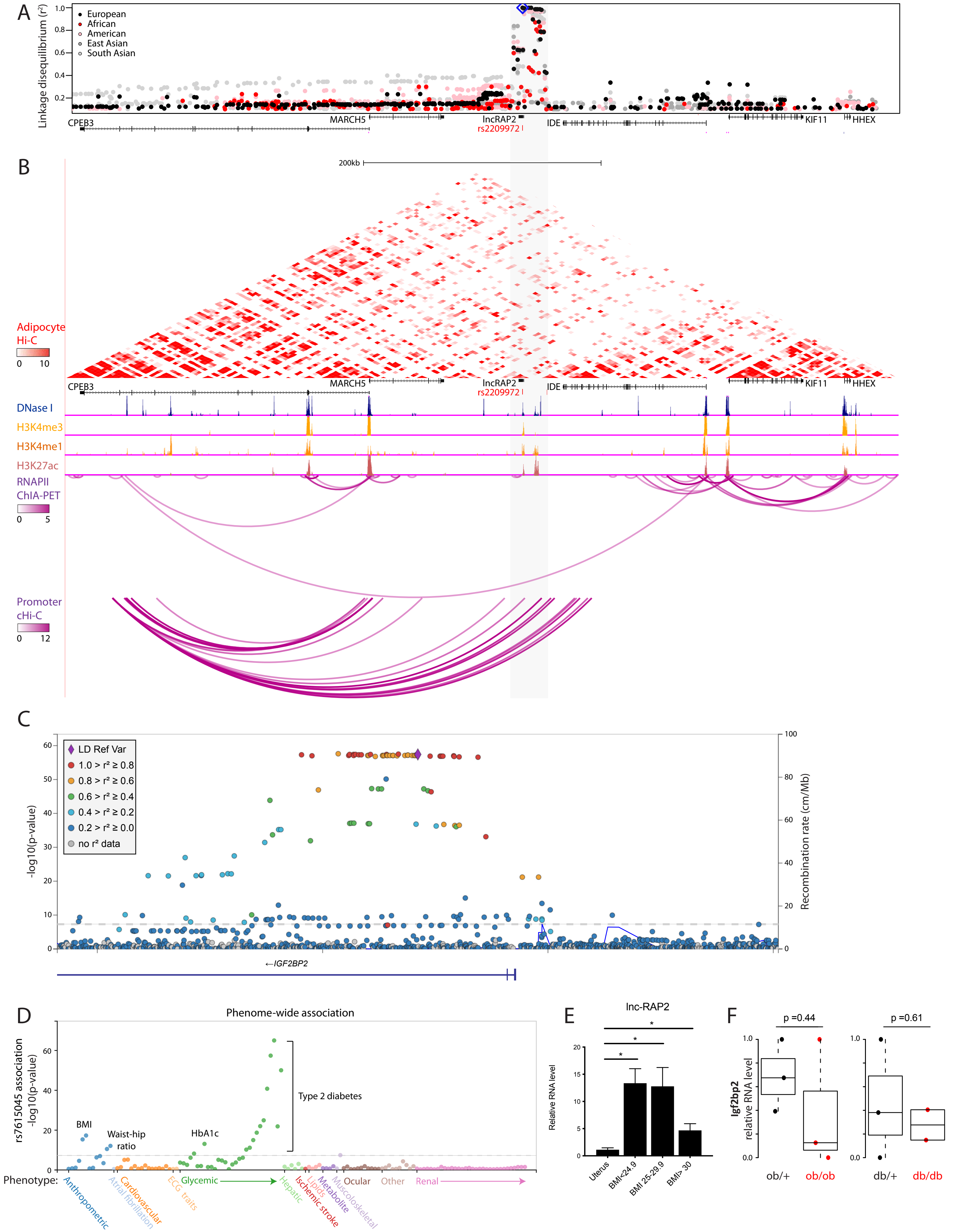
A
Linkage disequilibrium (r²)
1.0
0.8
0.6
0.4
0.2
European
African
American
East Asian
South Asian
CPEB3
MARCH5
lncRAP2
rs2209972
IDE
KIF11
HHEX
B
200kb
Adipocyte Hi-C
0
10
DNase I
H3K4me3
H3K4me1
H3K27ac
RNAPII ChIA-PET
0
5
Promoter cHi-C
0
12
C
LD Ref Var
1.0 > r² ≥ 0.8
0.8 > r² ≥ 0.6
0.6 > r² ≥ 0.4
0.4 > r² ≥ 0.2
0.2 > r² ≥ 0.0
no r² data
-log10(p-value)
Recombination rate (cm/Mb)
←IGF2BP2
D
Phenome-wide association
rs7615045 association -log10(p-value)
BMI
Waist-hip ratio
HbA1c
Type 2 diabetes
Phenotype:
Anthropometric
Atrial fibrillation
Cardiovascular
ECG traits
Glycemic
Hepatic
Ischemic stroke
Lipids
Metabolite
Muscoloskeletal
Ocular
Other
Renal
E
lnc-RAP2
Relative RNA level
Uterus
BMI<24.9
BMI 25-29.9
BMI> 30
F
Igf2bp2 relative RNA level
p =0.44
p =0.61
ob/+
ob/ob
db/+
db/db

**Figure S5. lncRAP2-Igf2bp2 genetic and expression variability are associated with obesity-linked diabetes risk. Related to Figure 4.**

(**A**) Human rs2209972 is in strong linkage disequilibrium with genetic variants within lncRAP2 but no other loci within 500kb. Linkage disequilibrium with rs2209972 (diamond) based on 1000 Genomes (Genomes Project et al., 2015) data for the indicated populations.

(**B**) Human rs2209972 maps to a lncRAP2 interaction domain. Heatmap displays human white adipocyte chromatin conformation by Hi-C (Paulsen et al., 2019). Tracks below display open chromatin and histone mark sequencing studies, and chromatin interactions involving RNA polymerase II or captured promoter regions (Table S4).

(**C**) Regional association with type 2 diabetes at the IGF2BP2 locus. Significance of association between genetic variants surrounding the IGF2BP2 transcription start site and type 2 diabetes in 898,130 European-descent individuals (Mahajan et al., 2018). Variants are colored based on 1000 Genomes All samples (Genomes Project et al., 2015) linkage disequilibrium with rs7615045 (diamond).

(**D**) IGF2BP2 rs7615045 is specifically associated with body mass, glycated hemoglobin (HbA1c), and type 2 diabetes. Phenome-wide association results between rs7615045 and 317 phenotypes across 16,278,030 individuals of various ancestries from the Type 2 Diabetes Knowledge Portal (http://www.type2diabetesgenetics.org/variantInfo/variantInfo/rs7615045). Only significant ($p < 0.05$) associations are shown.

(**E**) Adipose lncRAP2 is reduced with obesity. Shown are relative lncRAP2 levels (normalized to uterus, left) in adipose tissue samples pooled from patients with different body mass index (BMI) (Table S3).

(**F**) *Igf2bp2* is suppressed during diabetes onset and progression. *Igf2bp2* levels in visceral adipose tissue from leptin-deficient (ob/ob) and leptin receptor-deficient (db/db) 9-10 week-old mice (Wang et al., 2012a).

**Table S1:** Primer and DsiRNA sequences related to Key Results Table

| Primer mouse | forward 5´- 3´ | reverse 5´- 3´ | Source | Identifier |
|---|---|---|---|---|
| lnc-RAP-2 | GGTCCTTAGGCAGAGTCTTG | TCCATGGAGCACAATAGCTG | doi: 10.1073/pnas.1222643110. | IDT |
| 18S | GTAACCCGTTGAACCCCATT | CCATCCAATCGGTAGTAGC | doi: 10.1038/ncb2286. | IDT |
| 47s pre rRNA | CTCCTGTCTGTGGTGTCCAA | TGATACGGGCAGACACAGAA | This paper | IDT |
| AdipoQ | CGATTGTCAGTGGATCTGACG | CAACAGTAGCATCCTGAGCCCT | doi: 10.1038/ncb2286. | IDT |
| Cebpalpha | TGCGCAAGAGCCGAGATAAA | CCTTCTGTTGCGTCTCCACG | doi: 10.1038/ncb2286. | IDT |
| Fabp4 | ACAAGCTGGTGGTGGAATGTG | CCTTTGGCTCATGCCCTTT | doi: 10.1038/ncb2286. | IDT |
| Ppargamma | GTGCCAGTTTCGATCCGTAGA | GGCCAGCATCGTGTAGATGA | doi: 10.1038/ncb2286. | IDT |
| Glut4 | CTGTCGCTGGTTTCTCCAACT | CCCATAGCATCCGCAACATA | doi: 10.1038/ncb2286. | IDT |
| lnc-FIRRE | AGCCATTTTGAAGCAGGGAA | CTCTGAAGGGTCAGGTGATG | doi: 10.1038/nsmb.2764. | IDT |
| IGF2bp2 | GACTACCCCGACCAGAACTG | GAGGCGGGATGTTCCGAATC | This paper | IDT |
| IGF2 | GTGCTGCATCGCTGCTTAC | ACGTCCCTCTCGGACTTGG | This paper | IDT |
| Pgc1alpha | CCCTGCCATTGTTAAGACC | TGCTGCTGTTCCTGTTTTC | doi: 10.1038/ncb2286. | IDT |
| Cidea | TGCTCTTCTGTATCGCCCAGT | GCCGTGTTAAGGAATCTGCTG | doi: 10.1038/ncb2286. | IDT |
| Elovl3 | TTCTCACGCGGGTTAAAAATGG | GAGCAACAGATAGACGACCAC | This paper | IDT |
| 5´RACE g.s. RAP2 5´ | TTTCGCTTTGCACAAACATC | 0 | This paper | IDT |
| 5´RACE g.s. RAP2 inner | TCCACATTGCAATTCCTTTCT | 0 | This paper | IDT |
| 5´RACE g.s. RAP2 outer | TGGACTTCAGGGCCTCAGT | 0 | This paper | IDT |
| 3´RACE g.s. RAP2 outer | TGGAGTAAGGAATTTGCACCT | 0 | This paper | IDT |
| 3´RACE g.s. RAP2 inner | CCTCCTCCTTTTCCTCTTCC | 0 | This paper | IDT |
| M13 | GTAAAACGACGGCCAG | CAGGAAACAGCTATGAC | Thermo Fisher Scientific | K4575J10 |
| 0 | 0 | 0 | 0 | 0 |
| Primer human | forward 5´- 3´ | reverse 5´- 3´ | Source | Identifier |

| | | | | |
|---|---|---|---|---|
| hulncRAP-2 | TAAATACTGCAGGCGAAGGAC | CCTTCTACCCGCGTCATCA | This paper | IDT |
| human18S | GTAACCCGTTGAACCCCATT | CCATCCAATCGGTAGTAGCG | This paper | IDT |
| 0 | 0 | 0 | 0 | 0 |
| DsiRNAs | forward 5´- 3´ | reverse 5´- 3´ | Source | Identifier |
| DsiRNA lncRAP-2 #1 | CTGCATGGATGTTTTGTTTAGTCAA | AACTGATTTGTTTTGTAGGTACGTCCT | This paper | IDT |
| DsiRNA lncRAP-2 #2 | CTTCTACAAGAAATCAGAAACAGTA | ATGACAAAGACTAAAGAACATCTTCAG | This paper | IDT |
| DsiRNA Igf2bp2 #1 | AAGCAGTGCTCTGAATATCATACCA | TGGTATGATATTCAGAGCACTGCTTTG | mm.Ri.Igf2bp2.13.1 | IDT |
| DsiRNA Igf2bp2 #2 | AAGAGGCAAAGCTAGCTATTGAGAA | TTCTCAATAGCTAGCTTTGCCTCTTCT | mm.Ri.Igf2bp2.13.2 | IDT |
